# Enhanced adenoviral reactivity in Guillain-Barré syndrome after SARS-CoV-2 infection and vaccination

**DOI:** 10.1093/brain/awaf376

**Published:** 2025-10-07

**Authors:** Roberto Bellanti, Ana Candalija Iserte, Claire Bergstrom Johnson, John Goodfellow, Marina Johnson, Wanwisa Dejnirattisai, Stephen Keddie, Joseph J Campo, Gavin Screaton, David Goldblatt, Michael P Lunn, Alexander J Davies, Simon Rinaldi

**Affiliations:** Nuffield Department of Clinical Neurosciences, University of Oxford, Oxford OX3 9DU, UK; Department of Neuromuscular Diseases, Queen Square Institute of Neurology, University College London, London WC1N 3BG, UK; Nuffield Department of Clinical Neurosciences, University of Oxford, Oxford OX3 9DU, UK; Health and Biomedicine Department, Leitat Technological Center, Barcelona 08028, Spain; Nuffield Department of Clinical Neurosciences, University of Oxford, Oxford OX3 9DU, UK; Neuroimmunology Laboratory, Queen Elizabeth University Hospital, Glasgow G51 4TF, UK; Great Ormond Street Institute of Child Health, University College London, London WC1N 1EH, UK; Nuffield Department of Medicine, Wellcome Centre for Human Genetics, University of Oxford, Oxford OX3 7BN, UK; Division of Emerging Infectious Disease, Research Department, Faculty of Medicine, Siriraj Hospital, Mahidol University, Bangkok 10700, Thailand; Department of Neuromuscular Diseases, The Royal London Hospital, Barts Health NHS Trust, London E1 1BB, UK; Antigen Discovery, Inc., Irvine, CA 92618, USA; Nuffield Department of Medicine, Wellcome Centre for Human Genetics, University of Oxford, Oxford OX3 7BN, UK; Great Ormond Street Institute of Child Health, University College London, London WC1N 1EH, UK; Department of Neuromuscular Diseases, Queen Square Institute of Neurology, University College London, London WC1N 3BG, UK; Department of Neuroinflammation, National Hospital of Neurology and Neurosurgery, UCL Institute of Neurology, London WC1N 3BG, UK; Nuffield Department of Clinical Neurosciences, University of Oxford, Oxford OX3 9DU, UK; Nuffield Department of Clinical Neurosciences, University of Oxford, Oxford OX3 9DU, UK

**Keywords:** Guillain-Barré syndrome, COVID-19, vaccination, inflammatory neuropathy

## Abstract

Case reports and series suggested an association between SARS-CoV-2 and Guillain-Barré syndrome (GBS). However, the GBS epidemic which was predicted from early risk estimates did not materialize in overall case numbers, and no plausible mechanism for any link has been established. An increased risk of GBS following adenoviral vector-based COVID-19 vaccination has been more consistently demonstrated, but a pathophysiological explanation for this association has also not yet emerged. Here, we sought to identify whether patients with GBS following COVID-19 infection or vaccination had any distinct clinical or serological features differentiating them from one another or non-pandemic GBS, and to explore the potential mechanisms of any associations.

Between March 2020 and October 2021, sera from patients with GBS (*n* = 64) and controls (*n* = 70) were collected. Clinical features were retrieved from medical records. GBS cases were evaluated for diagnostic certainty by Brighton criteria and classified as non-COVID-19 associated (GBS-NC, *n* = 20), GBS after COVID-19 infection (GBS-AC, *n* = 10), or GBS after COVID-19 vaccination (GBS-AV, *n* = 34). The humoral responses to SARS-CoV-2 proteins and putative peripheral nerve antigens, and the cytokine profile of each group were established and compared. Antibodies cloned from the acute-phase plasmablasts of an individual with GBS-AC were also assessed for reactivity against SARS-CoV-2 and peripheral nerve antigens. Sera from GBS patients and from individuals who received COVID-19 vaccinations (*n* = 36: 16 ChAdOx1, 10 Ad26.COV2.S/Janssen and 10 tozinameran/Pfizer–BioNTech) without developing GBS were tested for IgG reactivity against SARS-CoV-2 and adenoviral proteins.

There were no clinical differences between the GBS groups. Patients with GBS-AC had a greater IgG reactivity to the S1 component of the SARS-CoV-2 spike protein compared to non-GBS COVID-19 controls. A minority of antibodies from cloned plasmablasts targeted SARS-CoV-2 proteins but there was no reactivity or cross reactivity with peripheral nerve antigens or tissue. There were no other serological or immunological differences between the GBS groups. However, when compared to uncomplicated vaccine recipients, GBS patients *in toto*, and each group individually, demonstrated significantly greater antibody reaction to a range of human adenoviral proteins.

Compared to controls exposed to the same immunological stimulus, antibody reactivities to viral antigens are enhanced in patients with GBS. However, we found no mechanistic link between S1 and peripheral nerve reactivity or pathology. Serological responses to adenoviral proteins may be directly involved in the pathogenesis of Guillain-Barré syndrome, potentially contributing to cases with currently unexplained aetiology.

## Teaser

Bellanti *et al.* find that patients with Guillain-Barré syndrome (GBS) show heightened antibody responses to human adenoviral proteins compared with controls. This suggests that adenoviruses may be causally associated with GBS, offering a possible explanation for cases with previously unclear aetiology.

## Introduction

During the early stages of the COVID-19 pandemic in 2020, numerous cases of the acute inflammatory polyradiculoneuropathy Guillain-Barré syndrome (GBS) occurring shortly after SARS-CoV-2 infection were reported. However, the substantial increase in GBS incidence predicted by risk estimates of initial case series^1,2^ did not emerge. Indeed, the billions of subsequent infections were associated with an overall decline of GBS in population-based studies.^3,4^ In 2021, a small spike in GBS was identified in surveillance studies and was temporally linked to first doses of the adenoviral vector-based COVID-19 vaccines [ChAdOx1 in the UK and Janssen (JNJ-78436735) in the USA], but not to first doses of the Pfizer–BioNTech mRNA-based vaccine or to second doses of any vaccine.^5,6^ A comparative, self-case-controlled study estimated a risk of one excess GBS diagnosis for every 68 809 infections or every 261 766 ChAdOx1 vaccinations; however, this study used post discharge coding data which can generate significant inaccuracies. A pair of nationwide UK studies found no quantifiable link of GBS to COVID-19 infection but a similar and more clearly established vaccination risk of 5.7 excess cases per million ChAdOx1 first doses.^3,5,7^ For comparison, GBS complicates around one in every 1000 *Campylobacter jejuni* infections.

The best explained mechanism of GBS to date is that of molecular mimicry between the *C. jejuni* bacterial surface lipo-oligosaccharide (LOS) and host gangliosides: LOS antigens generate cross-reactive anti-ganglioside antibodies that bind to structurally identical glycans present on peripheral nerve ganglioside species, resulting in complement fixation and dysfunction and/or destruction of peripheral axons.^8,9^ The mechanism leading to GBS following other prodromal triggers or infections, such as Zika virus (ZIKV),^10^ may be different and remains unclear, although virus-associated T-cell mechanisms have recently been proposed.^11^

In this study, we sought to establish whether patients with GBS following COVID-19 infection or vaccination have distinct clinical or serological features, and to explore the potential mechanisms of these associations.

## Material and methods

### Participants and sample collection

Between March 2020 and October 2021, serum samples were obtained from patients with GBS presenting to the John Radcliffe Hospital, Oxford, as well as from patients referred to the UK paranodal antibody testing service with a reported diagnosis of GBS. Clinical data paired with the samples were collected in a structured questionnaire. An additional cohort of serum samples were obtained from the West of Scotland Joint Neuroimmunology and Immunology Biobank established at the Queen Elizabeth University Hospital in Glasgow, Scotland. These samples were collected from patients with GBS in the acute phase, where a GBS diagnosis had been confirmed by standard clinical and neurophysiological criteria, following recent SARS-CoV-2 vaccination. Control samples were obtained from three groups. These were patients with chronic neuropathies (collected during the same time period, but from patients whose peripheral neuropathies were unequivocally known to have started before March 2020), patients with uncomplicated, convalescent COVID-19, and healthy individuals with no recorded history of prior infection with SARS-CoV-2 (and confirmed negative SARS-CoV-2 serology using the UK national testing platform) who were participating in the Oxford Protective T-cell Immunology for COVID-19 (OPTIC) project. Rates of SARS-CoV-2 immunity in these GBS and chronic neuropathy groups between 2 March and 23 July 2020 were compared to those in the contemporaneous wider population using the UK national serology platform.^12^

**Figure 1:**
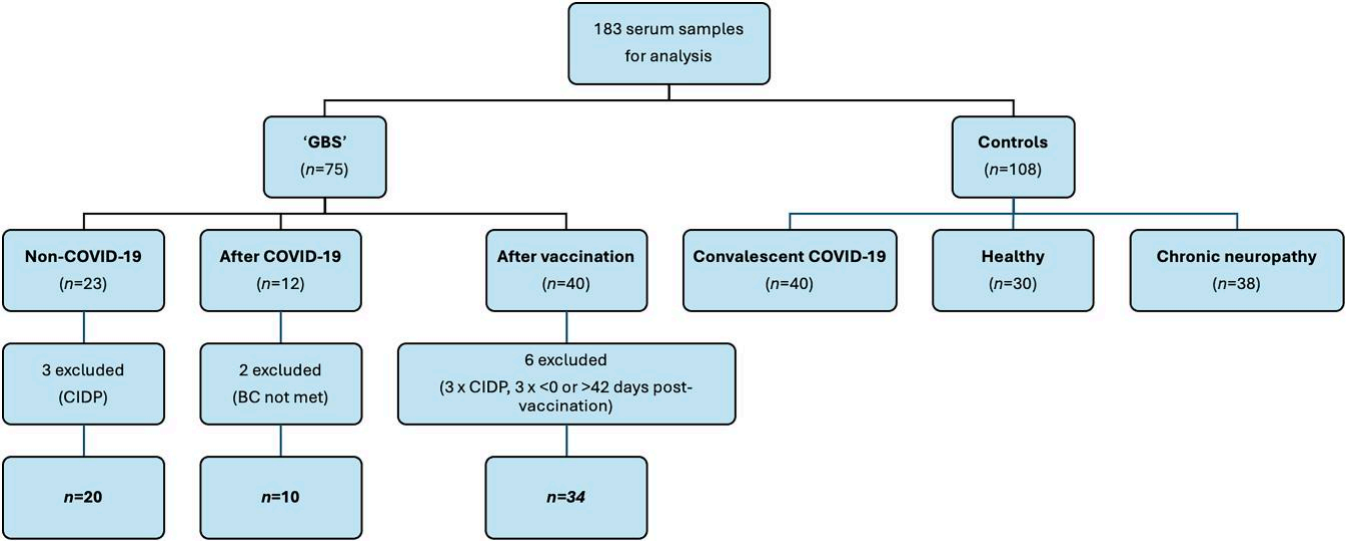
**Diagram of subjects included in and subsequently excluded from the study analysis**. BC = Brighton Collaboration; CIDP = chronic inflammatory demyelinating polyradiculoneuropathy.

During the course of the study, 75 patients with suspected GBS were identified from our hospital, from the Scottish sera or from sera sent for paranodal testing. Twenty-three were associated with neither COVID-19 infection nor vaccination (GBS-NC), 12 were associated with COVID-19 infection (GBS-AC) and 40 followed COVID-19 vaccination (GBS-AV) (Fig. 1 and Table 1). To be classified as GBS-AC, a positive PCR or serological test for SARS-CoV-2 was required at the time of GBS presentation or in the 42 previous days. Patients were classified as GBS-AV if they had received a SARS-CoV-2 vaccination in the 42 days prior to their GBS presentation. A total of 11 patients were excluded from further analysis as six GBS-NC cases were eventually diagnosed with chronic inflammatory demyelinating polyradiculoneuropathy (CIDP), two patients did not fulfil Brighton Collaboration certainty criteria for GBS,^13^ and three were excluded from GBS-AV group having developed GBS before their vaccination or more than 42 days afterwards (Fig. 1 and Supplementary Table 1).

**Table 1:** Demographics and clinical characteristics

	GBS-NC	GBS-AC	GBS-AV	Convalescent COVID-19 controls	Healthy controls	*P*-value
*n*	20	10	18^a^	40	30	
Median age, years	56.5	56.5	60	37.5	40	*P* < 0.01 (GBS-NC, GBS-AC and GBS-AV versus controls)
Gender	–	–	–	–	–	–
Males	13/20	10/10	11/18	19/40	15/30	–
Females	7/20	0/10	7/18	21/40	15/30	–
Male:female ratio	1.8:1	1:0	1.6:1	1:1.1	1:1	*P* = 0.04
Median interval between presumptive trigger and onset, days	8.5	9.5	10	–	–	–
Median nadir disability, mRS	5	5	4	–	–	*P* = 0.04 (GBS-NC versus GBS-AV)
Median Brighton Collaboration Criteria score	2	2	2	–	–	–
Electrophysiology	–	–	–	–	–	–
Axonal	3/20	3/10	0/16	–	–	–
Demyelinating	8/20	2/10	9/16	–	–	–
Normal/equivocal	9/20	5/10	7/16	–	–	–
Neuropathic pain	6/20	2/10	10/18	–	–	–
Cranial nerve involvement	12/20	6/10	8/18	–	–	–
Autonomic dysfunction	8/20	1/10	6/18	–	–	–
Respiratory failure	9/20	4/10	5/18	–	–	–
Median CSF protein, g/l	0.94	0.92	0.82	–	–	–

Number of patients per Guillain-Barré syndrome (GBS) group only includes those who met GBS Brighton Collaboration diagnostic criteria. Axonal electrophysiology defined as reduced or absent sensory nerve action potentials or compound muscle action potentials with mild slowing of conduction velocity and normal or marginally prolonged distal motor latencies. Demyelinating electrophysiology defined as prolonged distal motor latencies with significant slowing of conduction velocity, with or without conduction block. ‘Equivocal’ electrophysiology includes studies where inexcitable nerves were found. AC = after COVID-19 infection; AV = after COVID-19 vaccination; mRS = modified Rankin score; NC = non-COVID-19 associated.

^a^The post-vaccination GBS group includes patient cohorts from Oxford and Glasgow. In compliance with ethics requirements and limitations, we were not in possess of clinical data pertaining to the Glasgow cohort (*n* = 16), which was not included in this table. Of the 24 Oxford post-vaccine GBS cases, six were excluded (Fig. 1). Therefore, the total number of post-vaccine GBS patients with clinical data was 18. However, CSF and neurophysiology results were not available for one case, and in another neurophysiology was not done, hence 16 as total denominator in the electrophysiology subgroup.

Serum samples for a further 38 patients whose chronic neuropathies began before March 2020 were also analysed. This group included a high proportion of patients with previously identified nodal/paranodal antibodies who had had serum sent for repeat testing. Furthermore, 70 controls (40 convalescent COVID-19 and 30 healthy) were identified and sera collected. Control sera were also obtained from 36 individuals with uncomplicated COVID-19 vaccination who had participated in a previous study.^14^ All samples were collected following the first dose of an uncomplicated COVID-19 vaccination, including 16 recipients of the ChAdOx1 vaccine, 10 of the Janssen vaccine and 10 of the Pfizer–BioNTech vaccine.

The UK national serology platform^15^ was used to compare the frequency of serological evidence of prior SARS-CoV-2 infection in patients newly diagnosed with GBS during the first 4 months of the pandemic, with patients with chronic neuropathies whose disease onset unequivocally predated the start of the pandemic, and the background rate in England over the same period. This platform measured IgG responses to the SARS-CoV-2 trimeric spike protein using an ELISA developed by the University of Oxford, which was later commercialized as the Thermo Scientific Omnipath™ Combi SARS-CoV-2 IgG ELISA.

### Ethics

GBS serum samples were collected as part of the project ‘Investigating biomarkers, pathophysiology, and outcome in inflammatory and non-inflammatory neuropathies’ under ethical approval reference 14/SC/0280 or obtained from the West of Scotland Joint Neuroimmunology and Immunology biobank, ethics approval reference 21/WS/0070. Convalescent COVID-19 and non-infected healthy controls were collected as part of the OPTIC study under ethical approval reference 16/YH/0247.

### ELISA and cell-based assays

ELISAs were used to detect serum IgG reactivity against four SARS-CoV-2 proteins: spike protein S1 (Abcam, ab275927), spike protein S2 (Abcam, 274366), membrane protein M (MRC pure reagents, DU67699) and envelope protein E (Invitrogen, RP-87682). Nunc MaxiSorp 96-well plates were coated with proteins S1/S2/M (1 µg/µl in individual wells) or E protein (2 µg/µl) in PBS (50 µl/well) overnight at 4°C. Following three washes in 0.01% Tween-20/PBS, a blocking solution containing 5% milk, 0.05% Tween-20 and PBS was added for 1 h at room temperature (RT). Fifty microlitres per well of each serum diluted 1:100 in 1% milk/PBS was added and incubated for 1 h at RT. After five washes in 0.01% Tween-20/PBS, anti-human IgG-Fc-HRP secondary antibodies (Cat. No. A0170, Sigma) (1:3000) were added for 1 h at RT, then washed five times with 0.01% Tween-20/PBS. O-phenylenediamine dihydrochloride (OPD) (SigmaFast, Cat. No. P9187, Sigma-Aldrich) was added for 20 min for colourimetric development. The reaction was stopped with H_2_SO_4_ (4N) (25 µl) and absorbance at 492 nm read on a plate reader (FLUOstar OMEGA, BMG).

Ganglioside antibody ELISAs were used to identify serum reactivity against any of six common peripheral nerve gangliosides: GM1, GM2, GD1a, GD1b, GT1b and GQ1b. GM1 (G9652, Sigma), GM2 (G8397, Sigma), GD1a (G2392, Sigma), GD1b (1501, Matreya), GT1b (G2392, Sigma) and GQ1b (ALX-302-012-MC05, Enzo), as previously described.^16^

A cell-based assay was used to screen the sera for the presence of the paranodal and nodal antibodies to neurofascin 155 (NF155), NF186, NF140, contactin-1 (CNTN-1) and contactin-associated protein-1 (Caspr-1), as previously described.^17^

### Myelinating sensory neuron co-cultures

Myelinating co-cultures were prepared using human induced pluripotent stem cell (hiPSC)-derived sensory neurons and neonatal rat Schwann cells as previously described.^18,19^ hiPSC were differentiated to sensory neurons using a combination of small-molecule mediated dual-SMAD inhibition and WNT activation according to a previous protocol.^17^

### Assessment of complement-induced demyelination

Assessment of demyelination in live myelinated co-cultures was performed as previously described.^10^ Duplicate co-cultures on 13 mm diameter coverslips were treated with GBS or healthy control sera (1:50 dilution) in N2 ‘Complete’ media supplemented with 1% BSA and huNGF (25 ng/ml) for 1 h at 37°C (250 μl per well). Normal human serum (NHS) from a female donor in her fourth decade was added to one of duplicate wells (62.5 μl) to a final concentration of 20% NHS and incubated 24 h at 37°C. N2 ‘Complete’ media only was added to control wells. Wells were washed four times with PBS and fixed with 4% paraformaldehyde for 30 min at RT. Cells were immunostained for human IgG, as well as myelin basic protein (MBP) and NF200, as described.^10,18^ For quantification of demyelination, coverslips were mounted on glass slides for confocal imaging. A pre-set 5 × 5 grid of positions (or 7 × 7 for sparsely myelinated coverslips) at ×20 magnification (0.5 digital zoom) was used for automated acquisition across each coverslip. NF200 (405 nm, blue) and MBP (555 nm, red) were set to multitrack for simultaneous acquisition. Between five and seven 3-μm *z*-sections were collected to ensure cell culture features were imaged at all points along each coverslip. Images at each position were exported as maximum projections and used for counting intact and fragmented internodes in each field of view. The quantification of myelin feature number and size was performed on the thresholded MBP signal. The Analyse Particle function in ImageJ was used to count and measure the size of myelin features per region of interest (ROI; 25–49 ROIs per coverslip); myelin features were defined within lower (5 μm^2^) and upper (infinite) size limits. Average myelin features size was calculated as: total myelin area/number of features. Smaller values indicate greater fragmentation of myelin.

### Plasmablast isolation and monoclonal antibody cloning

Peripheral blood mononuclear cells (PBMCs) were obtained from a single individual 19 days after COVID-19 infection, and 4 days into the development of subsequently confirmed GBS. Acute-phase plasmablasts (CD19+, CD3−, CD20−, CD27++, CD38++) were singly sorted, and their heavy and light chains cloned into expression vectors using reverse transcriptase followed by nested PCR.^20^ The monoclonal antibodies generated were then assessed for reactivity against SARS-CoV-2 antigens, peripheral nerve gangliosides, nodal/paranodal proteins and myelinating co-cultures as above.

### Cytokine multiplex assay

A U-PLEX Biomarker Group 1 (Human) electroluminescence-based multiplex assay (Cat. No. K15067L-1, Mesoscale Diagnostics) measured levels of the cytokines IL1b, IL2, IL4, IL6, IL8, IL12p70, IL17a, IFNγ, TNFα and MIP-1a in all GBS and control sera. Serum samples were analysed neat (except single sample diluted 1:1 in PBS with subsequent correction of measured concentration). The cytokine U-plex assay was run according to manufacturer’s instructions for the simultaneous detection of 10 inflammatory cytokines in a single 50 µl sample of patient or healthy control serum. Briefly, cytokine-specific biotinylated capture antibodies were pre-incubated with one of 10 individual linkers and bonded with a 10-spot U-plex plate for 1 h at RT on a microplate shaker (700 rpm). After washing in PBS containing 0.05% Tween-20 (PBS-T), samples and calibration standards were added to the plate in triplicate, including blank control wells, and the plate was sealed and incubated 1 h at RT with shaking (700 rpm). After five washes in PBS-T, wells were incubated with a cocktail of cytokine-specific detection antibodies for 1 h at RT with shaking (700 rpm). After washing, MSD Gold Read buffer B (150 µl per well) was added and the plate read on an MSD instrument. Concentrations of each analyte in each well were interpolated from standard curves and average values obtained from sample duplicates. Samples were run across three separate plates, each with a full set of calibration standard curves.

### Adenoviral antigen array protocol

Multi-adenovirus proteome microarrays were fabricated containing 207 full-length or fragmented recombinant proteins representing the genes of human adenovirus (HAdV) serotypes 4 (*n* = 43), 5 (*n* = 39), 26 (*n* = 22), 40 (*n* = 36) and 41 (*n* = 34), chimpanzee adenovirus (ChAdV) serotype Y25 (*n* = 31), and SARS-CoV-2 N and S2 proteins (Supplementary Table 3). ChAdV-Y25 proteins were included as the ChAdOx1 vector was adapted from this virus. The S2 fragment of SARS-CoV-2 S was selected due to ease of expression and correlation with exposure on the proteome microarray.^21,22^ Genomic templates for HAdV 4 and 5 were acquired from BEI Resources, HAdV 40 and 41 from ATCC, and HAdV26 and ChAdV Y25 by synthesis at Twist Bioscience. Each open reading frame (ORF) sequence was amplified by PCR and inserted into the vector pXT7 by recombination in *E. coli* to establish a library of partial or complete coding DNA sequences. Proteins were expressed using a coupled *E. coli* cell-free *in vitro* transcription and translation (IVTT) system (Rapid Translation System, Biotechrabbit, Cat. No. BR1400201) and spotted onto nitrocellulose-coated glass AVID slides (Grace Bio-Labs Inc.) using an Omni Grid Accent robotic microarray printer (Digilabs Inc.). Each expressed protein included a 5′ poly-histidine (His) epitope and 3′ haemagglutinin (HA) epitope. Proteome array chip printing and protein expression were quality checked by probing random slides with anti-His and anti-HA fluorescently labelled monoclonal antibodies and quantifying spot signals using a microarray scanner.

Serum samples were diluted (1:200) and incubated on the multi-adenovirus proteome chips overnight at 4°C on a rocker. Bound IgG was detected with DyLight650-conjugated goat anti-human IgG-Fc (Bethyl Laboratories, Cat. No. A80-104D5). Washed and dried Proteome chips were scanned, and the spot and background signal intensities (SI) were exported into the R package for statistical analysis. Spot SIs were adjusted for local background by subtraction, and values were floored to 1. Next, the data were normalized by dividing the protein spot values by the median of IVTT control spots (IVTT expression reactions with no adenovirus ORFs), and values were log transformed using the base-2 logarithm. Thus, normalized data represented the log_2_ signal-to-noise ratio, where a value of 0 represents specific antibody SI equal to the background, 1.0 represents twice the background, 2.0 represents 4-fold over background, and so forth. Adenovirus protein responses were classified as seropositive for SI of at least 1.0, or twice the background. A protein was classified as ‘reactive’ if at least 5% of study participants responded to the protein, i.e. SI > 1. Individual antibody responses or mean antibody responses were visualized using the ComplexHeatmap package.^23^

### Statistical analysis

Statistical analysis was performed in Prism v9.1.0 (GraphPad) and R (R Core Team, version 4.3.3, 2024). Statistical comparisons of the nadir disability and degree of diagnostic certainty among groups were performed using the Kruskal-Wallis/Dunn’s test. The proportions of clinical and neurophysiological subtypes of GBS, clinical features, SARS-CoV-2 antibodies, anti-ganglioside antibodies, paranodal/nodal antibodies and percentages of IgG reactivity were compared using contingency analyses with the application of a two-tailed Fisher exact test for individual group-group comparisons and the χ^2^ test for comparisons between all groups. Analysis of confocal microscopy images was performed by Mann-Whitney test for comparison of two groups and ANOVA for comparison of three or more groups, with correction for multiple comparisons where indicated. Differences in antibody levels on the proteome microarray platform were assessed using *t*-tests, and *P*-values were adjusted for the false discovery rate using the method described by Benjamini and Hochberg.^24^

## Results

Demographics, clinical features, cytokine profiles and humoral responses to SARS-CoV-2 proteins and putative peripheral nerve antigens were compared between the three GBS groups and controls (non-neurological convalescent COVID-19 and healthy). In the GBS-AC group, 10/12 patients (and 8/10 following exclusions) were COVID-19 seropositive. The two negative patients had both recently tested positive for SARS-CoV-2 by PCRs (Supplementary Table 1). This is similar to uncomplicated COVID-19, where 4/40 were seronegative despite all having had recent positive SARS-CoV-2 PCRs, and consistent with the results of a previous large scale study, which showed that 24% of patients with PCR-confirmed SARS-CoV-2 infections are ‘non-responders’ who do not develop anti-spike antibodies.^15^ The antibody reactivity to SARS-CoV-2 and adenoviral proteins was compared between GBS groups (individually and in combination) and uncomplicated recipients of the ChAdOx1, Janssen and Pfizer–BioNTech vaccines.

### Demographics

The median ages of the GBS-NC, GBS-AC and GBS-AV groups were not significantly different (Kruskal-Wallis; Table 1). The convalescent COVID-19 and healthy control groups were younger (median ages 37.5 and 40): this difference was statistically significant when compared to the GBS-AV and GBS-NC groups (*P* < 0.01, Kruskal-Wallis) and with the GBS-AC group (*P* < 0.05) (Table 1 and Supplementary Fig. 1A). The male-to-female ratio also differed significantly between groups (*P* = 0.04, chi-square), with proportionally more males in the GBS groups as expected (especially in GBS-AC where 10/10 were male) (Table 1 and Supplementary Fig. 1B).

### Clinical features

The median interval between the COVID-19 symptoms (9.5 days) or vaccination (10 days) and GBS onset was similar across the groups. A preceding infection was identified in only 4 of 23 GBS-NC cases, in these documented 8.5 days before GBS. The time from GBS onset to blood sample collection was different between GBS-AC and the GBS-AV groups (*P* = 0.0034) (Supplementary Fig. 2B). Blood samples were collected later in the GBS-AV group compared to the other GBS groups and to non-GBS COVID-19 controls (Supplementary Fig. 2C). In line with previous reports, all 34 included patients in the GBS-AV group had received a first dose of the adenoviral vector-based ChAdOx1 vaccine, whereas two of the six ultimately excluded from further analysis had received a Pfizer–BioNTech mRNA-based vaccine. The median nadir disability was significantly lower (better) in the GBS-AV (modified Rankin score, mRS = 4) compared to GBS-NC group (mRS = 5, *P* = 0.04). There was no significant difference in the Brighton Collaboration Criteria diagnostic certainty distribution (prior to exclusions), clinical or electrophysiological subtype (Supplementary Fig. 3A–D), or frequencies of neuropathic pain, cranial nerve (including facial nerve), autonomic and respiratory involvement, between any of the GBS groups (Supplementary Fig. 3E–H).

The frequency of serological evidence of SARS-CoV-2 infection in GBS and chronic neuropathy patients in the early phase of the pandemic was compared to national infection data obtained using the same testing platform. Between 2 March and 23 July 2020, significantly more patients newly diagnosed with GBS and meeting Brighton Collaboration criteria (levels 1–4, 33.3%, 6/18, or levels 1–2 only, 6/14, 42.6%) had evidence of prior SARS-CoV-2 infection compared to those with chronic neuropathies whose disease onset unequivocally predated the start of the pandemic [8.8% (3/34), *P* < 0.05, Fisher’s exact test], and the background rate in England over the same period (6.1%, 214/3084, *P* < 0.001, Fisher’s exact test), using data from the UK national serology testing platform (Supplementary Fig. 4). This could reflect the highly selective cohort of GBS samples included in this study, many of which were sent for testing as cases of GBS with known recent SARS-CoV-2 infection, the fact that patients with pre-existing neuropathy or other diseases were less COVID-19 exposed through shielding, or that population testing for COVID-19 antibodies lacked coverage and sensitivity in early 2020.

### Serological analyses

**Figure 2:**
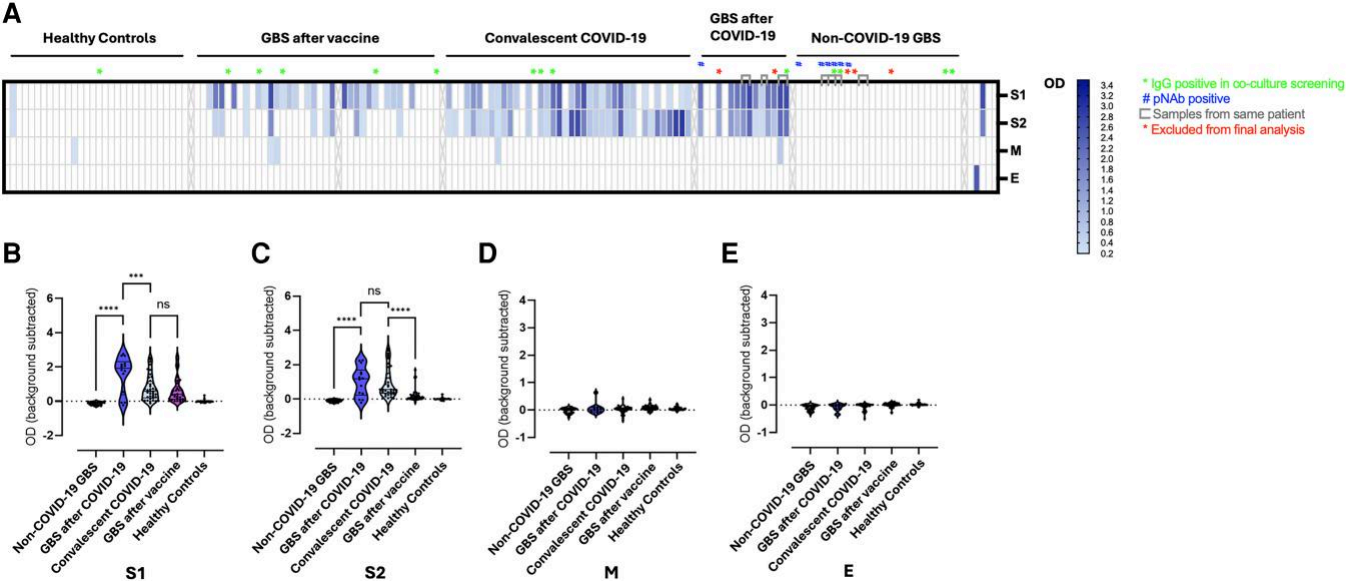
**IgG reactivity against SARS-CoV-2 proteins**. (**A**) ELISA IgG immunoreactivity to Sars-CoV-2 envelope (E), membrane (M) and spike (S1, S2) proteins in sera from GBS patients and controls. Green asterisks indicate IgG positive sera in co-culture screening. Blue hash marks indicate paranodal/nodal antibody positive sera. Colorimetric scale indicates background-subtracted optical density (OD) values. (**B**) Reactivity to S1 was higher in sera from patients with post-COVID-19 GBS compared to convalescent (non-neurological) COVID-19 and non-COVID GBS. (**C**) Reactivity to S2 protein was overall lower after COVID-19 vaccination compared to natural COVID-19 infection. (**D** and **E**) Reactivities to M and E proteins, respectively. ***P < 0.001; ****P < 0.0001; ns = not significant. GBS = Guillain-Barré syndrome.

We first confirmed SARS-CoV-2 immunity by testing all sera for IgG reactivity to viral envelope (E), membrane (M) and spike (S1, S2) proteins by ELISA. SARS-CoV-2 immunoreactivity was found only in sera from patients with previous COVID-19 infection or vaccination (Fig. 2A–E), including convalescent COVID-19 controls (35/40, 87.5%), GBS-AC (9/12, 75%) and GBS-AV (26/39, 66.7%). All healthy controls from the OPTIC study were negative. The most frequently identified SARS-CoV-2 antibodies were against S1 and S2 proteins. No samples were reactive against the E protein. The optical density (OD) of immunoreactivity to S1, as a measure of plate-bound IgG, was higher in sera from patients with GBS-AC compared to COVID-19 cases without neurological sequelae (*P* < 0.001), possibly suggesting a more pronounced or proximate humoral response to the virus in the GBS-AC group (Fig. 2B). A moderate positive correlation was found between time from infection or vaccination to blood sample collection and IgG reactivity to S1 by OD in the convalescent COVID-19 group, whereas the only positive correlation in GBS was with protein M in the GBS-AC group (Supplementary Fig. 5). Reactivity to the S2 protein was lower overall after COVID-19 vaccination compared to convalescent COVID-19 (*P* < 0.0001) and GBS after COVID-19 (*P* < 0.01) (Fig. 2C) as in previous studies.^25^ When comparing IgG reactivities between S1 and S2 proteins in each sample group, patients with GBS-AV also had overall higher S1 reactivity compared to S2 (S1 versus S2 OD, 0.42 versus 0.08, *P* = 0.0014) whereas reactivity to both spike proteins was similar in GBS-AC patients, (S1 versus S2 OD, 1.92 versus 1.18, *P* = 0.1781) as well as the convalescent COVID-19 group (S1 versus S2 OD, 0.58 versus 0.56, *P* = 0.2711).

Ganglioside antibodies were not statistically significantly more frequent in the GBS-NC group (7/20, 35%) compared to the GBS-AC (2/10, 20%) and GBS-AV (2/30, 6.7%) groups. There were fewer patients with IgG anti-ganglioside antibodies in the GBS-AV compared to GBS-NC [*P* = 0.01, OR 0.08 (95% CI 0.01 to 0.63); Supplementary Fig. 6A].

Live cell-based screening for any IgG reactive against known paranodal/nodal proteins was positive in 2 of 20 (10%) GBS-NC sera (both with pan-neurofascin antibodies), 1 of 10 (10%) GBS-AC (NF155 antibodies), and 8 of 38 (21%) patients with chronic neuropathy. No sera from GBS-AV, convalescent COVID-19 or healthy controls had nodal/paranodal antibody activity (Supplementary Fig. 6B). Whilst paranodal/nodal antibodies were more frequently identified in the GBS and neuropathy groups compared to non-neurological controls, as expected (chi-square test: *P* = 0.004), there was no significant difference between the GBS groups themselves.

**Figure 3:**
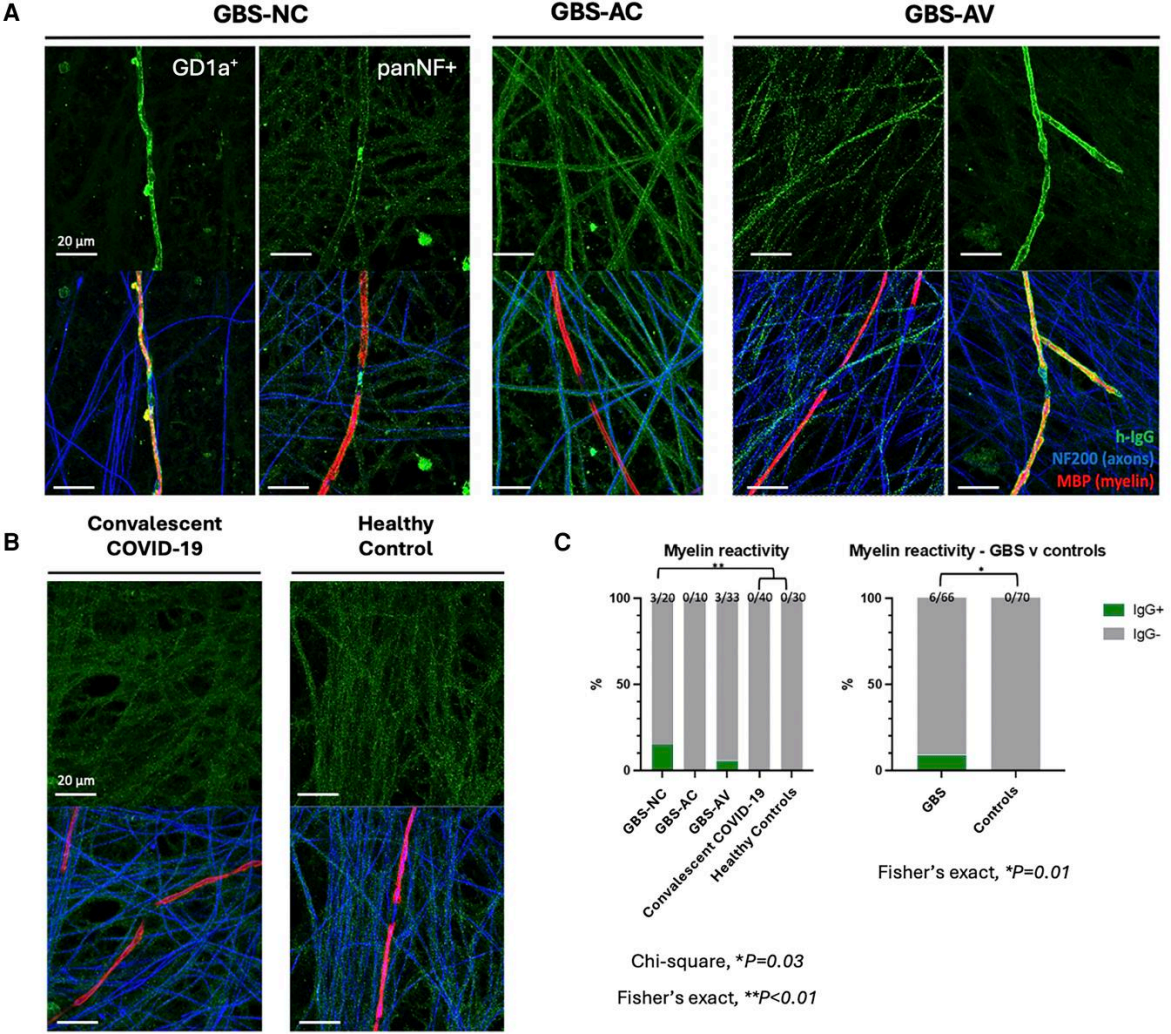
**Immunohistochemistry of myelinating co-cultures treated with serum from patient with GBS, convalescent COVID-19 controls and healthy controls**. (**A**) Cultures treated with sera from patients with non-COVID-19 GBS and GBS after vaccine showed IgG myelin-reactivity. (**B**) Myelin-reactivity was not observed in convalescent COVID-19 and healthy controls. (**C**) Myelin reactivity was higher in GBS sera when compared all together with controls (Fisher’s exact, *P* = 0.01). The non-COVID GBS samples with co-culture reactivity were also either GD1a or pan-neurofascin (panNF) seropositive, as indicated. All other samples with co-culture reactivity were glycolipid and paranodal antibody negative. AC = after COVID-19 infection; AV = after COVID-19 vaccination; GBS = Guillain-Barré syndrome; NC = non-COVID-19 associated.

**Figure 4:**
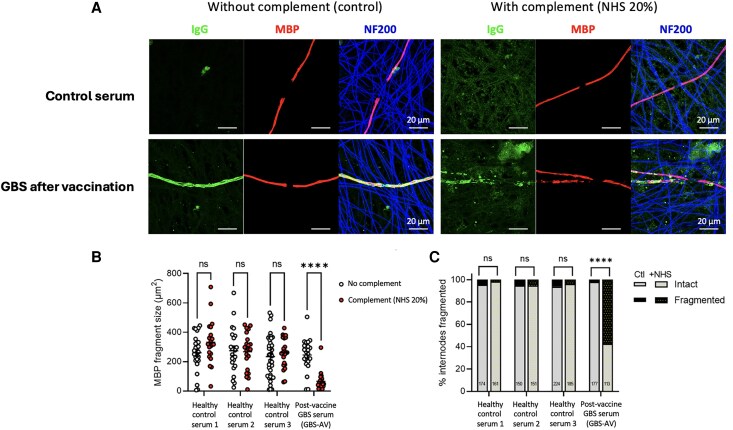
**Immunocytochemistry of myelinating co-cultures treated with control and patient (GBS after COVID-19 vaccine) sera**. (**A**) After adding serum from a patient with post-vaccine GBS, IgG myelin reactivity and complement-mediated demyelination were observed morphologically, and as evidenced by (**B**) smaller MBP fragment size and (**C**) higher percentage of internode myelin fragmentation (****P < 0.0001, Fisher’s exact test). Numbers in graphs indicate the total number of internodes in each sample. Scale bars as indicated. Ctl = control; GBS = Guillain-Barré syndrome; NHS = normal human serum.

IgG reactivity against any component of the myelinating co-culture system was observed in 3/20 (15%) GBS-NC sera, 1/9 (11%) GBS-AC, 5/31 (16%) GBS-AV, 5/38 (13%) of chronic neuropathy patients, 3/40 (7.5%) convalescent COVID-19 controls and 1/30 (3.3%) healthy sera, with no significant differences between groups (chi-square, *P* = 0.5914; Supplementary Fig. 6C). One GBS-AC and three GBS-AV samples were not tested due to limited sample volumes. However, IgG reactivity to myelin/myelinating Schwann cells was exclusively observed with sera from GBS-NC and GBS-AV patients (Figs 3 and 4A). Significantly more GBS sera displayed myelin reactivity than controls (Fisher’s exact, *P* = 0.01; Fig. 3C), but there was no significant difference between the different GBS groups. After incubation with GBS sera containing IgG strongly reactive to myelin, the addition of a source of human complement (20% normal human serum, NHS) resulted in myelin damage, which was not observed when NHS was added to a culture treated with control serum (Fig. 4A). Myelin damage was quantified by decrease in MBP fragment size (Fig. 4B) and higher percentage of MBP internode fragmentation (Fig. 4C).

### Post-COVID-19 GBS plasmablast specificities

**Figure 5:**
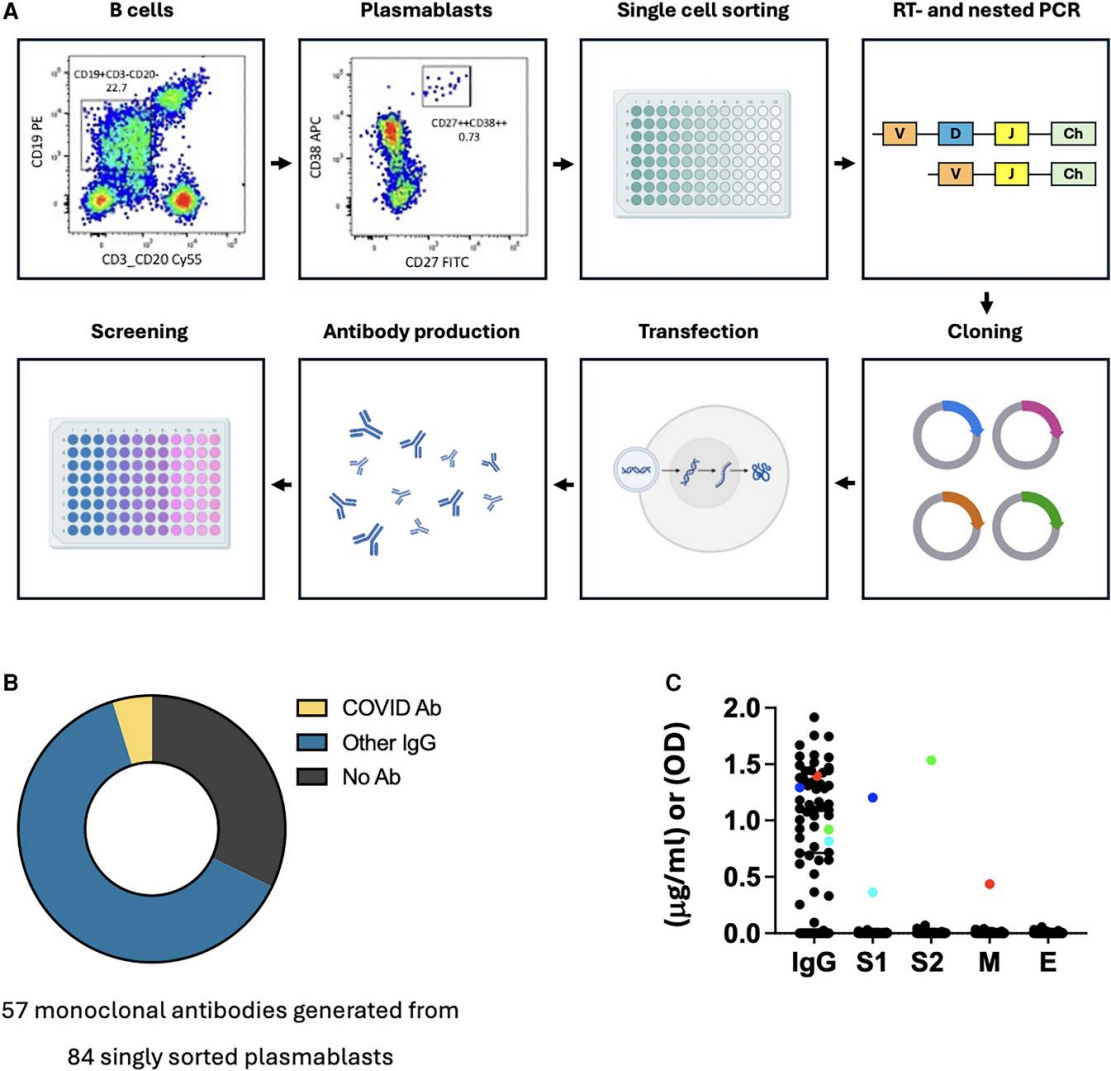
**Production and screening of monoclonal antibodies from a patient with GBS following Sars-CoV-2 infection**. Spike and membrane reactive antibodies did not cross-react with peripheral nerve antigens. Other cloned acute phase antibodies did not react with peripheral nerve. Fifty-seven monoclonal antibodies were generated from 84 singly sorted plasmablasts: of these, two (3.5%) reacted against the S1 component of the SARS-CoV-2 spike protein, one (1.8%) against the S2 component, and one (1.8%) to the membrane protein. No monoclonal antibodies reacted against the viral envelope. GBS = Guillain-Barré syndrome; OD = optical density.

To investigate the humoral response to SARS-CoV-2 infection for cross-reactivity to peripheral nerve antigens, we isolated and cloned monoclonal antibodies from the singly sorted, acute-phase plasmablasts of one COVID-19-GBS patient 19 days after infection and 4 days after GBS onset (Fig. 5). Of the 57 monoclonal antibodies generated from 84 singly sorted plasmablasts, two (3.5%) reacted against the S1 component of the SARS-CoV-2 spike protein, one (1.8%) against the S2 component and one (1.8%) to the membrane protein. No monoclonal antibodies reacted against the viral envelope. Neither the spike and membrane reactive antibodies, nor the other 53 antibodies whose specificity was not determined, cross-reacted with peripheral nerve antigens in the ganglioside ELISA, nodal/paranodal cell-based assay or in myelinating co-cultures. The original serum from the same patient also did not show reactivity to axonal or myelin peripheral nerve antigens.

### Cytokine profiling

We assessed the serum of all patients and controls using an electrochemiluminescent (ECL) cytokine profile array. Multiple comparison analysis revealed no differences between GBS-NC, GBS-AC, convalescent COVID-19 and heathy controls (Supplementary Fig. 7). In GBS as a whole, there was a trend for higher levels of IFN-y, IL-12p70, IL-1B, IL-2 IL-6 and IL-8, but this did not reach statistical significance.

### Adenoviral array analysis

**Figure 6:**
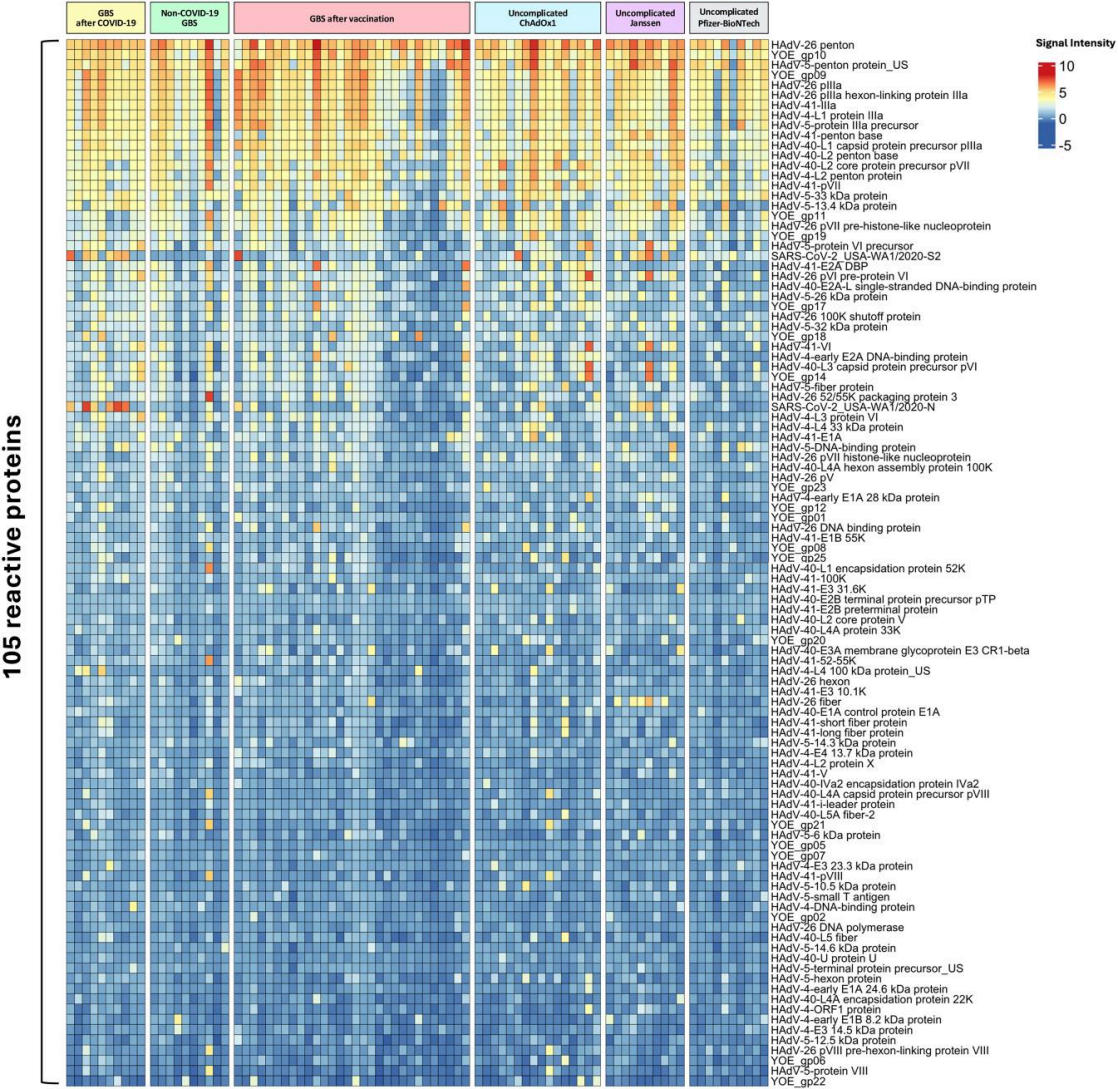
**Heat map of IgG antibody reactivity to 105 of 207 proteins probed**. Reactivity to adenoviral and SARS-Cov-2 proteins was compared across GBS and uncomplicated vaccine recipient groups. A 5% seroprevalence cut-off was applied. Seropositive cut-off = 1.0. GBS = Guillain-Barré syndrome.

We hypothesized that the adenoviral vector present in some COVID-19 vaccinations (ChAdOx1 and Janssen) might itself be immunogenic and provoke differential reactivity in some that might be associated with GBS. To evaluate this, sera from patients in all GBS and uncomplicated vaccine groups were analysed for IgG reactivity to SARS-CoV-2 and adenoviral proteins using antibody detection arrays. Reactivity was detected to 105 of 207 probed adenoviral and SARS-CoV-2 proteins, applying a 5% seroprevalence cut-off (Fig. 6).

**Figure 7:**
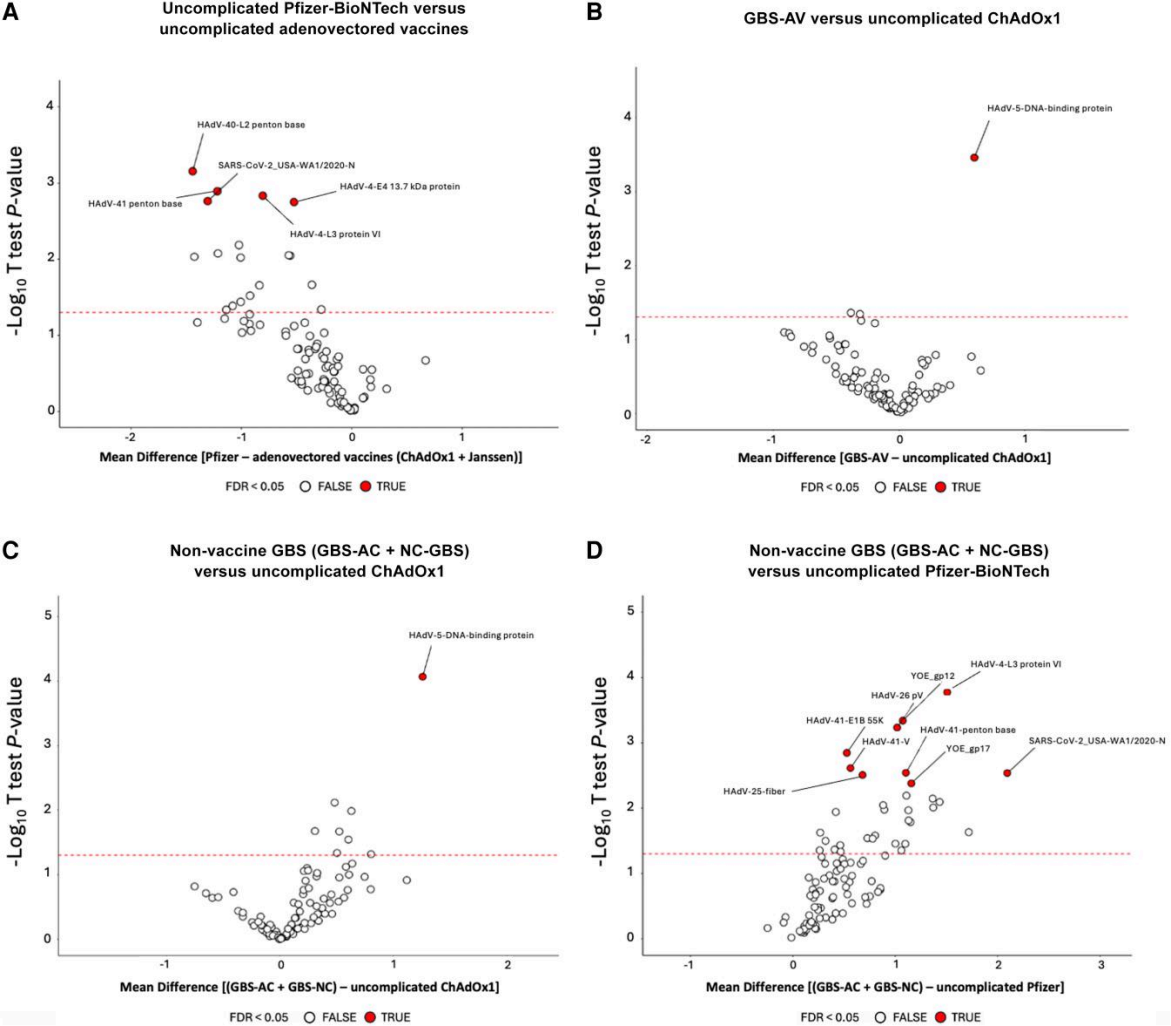
**Volcano plots of comparisons between uncomplicated vaccines and GBS patients**. (**A**) Comparison between uncomplicated Pfizer-BioNTech versus uncomplicated adenovectored vaccines (ChAdOx1 and Janssen together); (**B**) GBS-AV versus uncomplicated ChAdOx1 vaccine; (**C**) non-vaccine GBS (GBS-AC + NC−GBS) versus uncomplicated ChAdOx1 vaccine; and (**D**) non-vaccine GBS (GBS-AC + NC−GBS) versus uncomplicated Pfizer-BioNTech. *P* = 0.05. AC = after COVID-19 infection; AV = after COVID-19 vaccination; FDR = false discovery rate; GBS = Guillain-Barré syndrome; NC = non-COVID-19 associated.

We first compared reactivity amongst the GBS groups. As expected, there was higher reactivity to the SARS-CoV-2 S2 and N proteins in the GBS-AC group, and higher reactivity to S2 only in GBS-AV, when compared to GBS-NC, in keeping with recent COVID-19 infection or vaccination respectively. However, these comparisons did not remain significant when corrected for a false discovery rate of <0.05. There was no evidence of increased reactivity to any adenoviral antigens in GBS-AV when compared to all other GBS groups either assessed together or individually (Supplementary Table 2). However, there was evidence of increased reactivity to a range of adenoviral antigens in individuals who received COVID-19 vaccinations with an adenoviral vector compared to those receiving mRNA-based vaccinations (Fig. 7A). Furthermore, when GBS-AV sera were compared to those from individuals who received the same COVID-19 vaccination but did not develop GBS (‘uncomplicated ChAdOx1’), enhanced reactivity to Human Adenovirus Type 5 DNA binding protein (HAdV5-DNA-BP) was observed in those with GBS-AV (Fig. 7B and Supplementary Table 2). Enhanced reactivity to this target was also seen in non-vaccine associated GBS (GBS-NC and GBS-AC) when compared to uncomplicated ChAdOx1 vaccination (Fig. 7C). Furthermore, when comparing non-vaccine associated GBS against non-adenoviral vector vaccination controls (‘uncomplicated Pfizer-BioNTech’), a broad range of enhanced adenoviral reactivities were seen in the GBS groups, in the absence of exposure to an adenoviral vector (Fig. 7D and Supplementary Table 2). Taken together, these results demonstrate an immunological response to adenoviral antigens induced by adenoviral vector containing vaccinations, a differential response to adenoviral antigens in individuals who do versus those who do not develop GBS following ChAdOx1 vaccination, and a generally increased reactivity to some adenoviral antigens in GBS as a whole. To explore the potential confound of prior IVIg administration on these results, we compared the adenoviral reactivity in GBS serum samples taken following IVIg to those taken from GBS patients who had not yet received IVIg (Supplementary Fig. 8). In summary, there were no systematic changes in signal intensity across the array in post-IVIg versus non-IVIg treated samples, with most antigen spots producing similar signal intensity in both groups, some producing higher signal in post IVIg samples, and some producing higher signals in non-IVIg samples. The antigens producing the greatest differences between groups were not restricted to any particular virus, and the average signal intensity across all spots was no different between the groups. Furthermore, there were no significant signal intensity differences between the groups for any of the specific adenoviral antigens which produced differential responses in GBS versus uncomplicated vaccine controls (as per Fig. 7). As such, whilst IVIg could theoretically confound these results, we could find no evidence of such an effect in practice.

## Discussion

GBS is the leading cause of acute neuromuscular paralysis worldwide.^8,9^ Classically postinfectious, GBS is caused by an identifiable trigger (sometimes without an isolated organism) in about two-thirds of cases, whereas one-third remains unexplained, as does the repeatedly described seasonal variation in incident cases outside of Northern China.^8^ Evidence of a time-locked, measurable spike in GBS cases following vaccination with the adeno-vectored vaccines ChAdOx1 and Janssen, not shown with any of the other vaccines, implicated the adenoviral vector as a common causal factor.^5,6^ Our clinical and serological analysis investigated whether GBS associated with COVID-19 infection is immunologically different from non-COVID-19-related GBS, and whether GBS after vaccination shows any similarity or differences to either of the above. We used sample cohorts originally collected for other research purposes, as a prospective study was not planned, nor would now be possible.

We explored the serum reactivity profile by testing for antibodies usually associated with GBS and autoimmune nodopathies, inflammatory cytokines, and using arrays to assess reactivity differences to SARS-CoV-2 and adenoviral antigens. A major challenge in investigating the association with adenovirus is the high prevalence of adenovirus seroconversion in the general population due to recurrent annual infections, making it difficult to differentiate vaccine-induced responses from pre-existing immunity. Similarly, by the end of the COVID-19 pandemic, nearly the entire UK population had COVID-19 seroconverted, either through SARS-CoV-2 infection or vaccination. Consequently, variations in population characteristics and the semi-quantitative magnitude of anti-viral responses (measured in optical densities), may be among the few viable approaches to investigating these relationships. Acute SARS-CoV-2 infections, the acute nature of GBS, and the precise timing of vaccine administration—some with adenoviral vectors and some without—provided a unique opportunity to explore acutely enhanced immunological, and particularly adenoviral, antibody responses.

We found enhanced reactivity to S1 protein in patients with GBS following COVID-19 infection, compared to individuals with uncomplicated COVID-19, enhanced reactivity to HAdV5-DNA-BP in patients with GBS following ChAdOx1 vaccination, compared to individuals with uncomplicated ChAdOx1 vaccination, and more broadly enhanced adenoviral responses in the GBS cohort as a whole. However, we found no distinct clinical or serological features in patients with GBS following SARS-CoV-2 infection or vaccination compared with ‘sporadic’, non-COVID-19-associated GBS. The frequency of peripheral nerve-reactive antibodies was similar between GBS groups, and serological findings were heterogeneous within groups. Furthermore, antibodies cloned from the acute-phase plasmablasts of a patient with post-COVID-19 GBS targeted SARS-CoV-2 proteins without showing reactivity or cross-reactivity to peripheral nerve axolemmal or myelin antigens. Whilst not confirming that GBS, GBS after COVID-19, and GBS after vaccination are the same entity, our analysis found no distinguishing features to differentiate between them. On the other hand, unequivocally identifying only patients whose GBS was caused by SARS-CoV-2 infection or vaccination, rather than simply coincidental to these events, is challenging. This potential confound would reduce the likelihood of detecting a distinct serological signature in GBS-AV patients, yet despite this, the adenoviral association persists at the group level.

The prior administration of IVIg represents a potential confound when interpreting IgG reactivity in serum, particularly in diseases like GBS, where treatment is frequently initiated early. Exogenous immunoglobulins could theoretically contribute to ‘false positive’ results through passive transfer of reactive IgG, while also potentially causing ‘false negatives’ by saturating the neonatal Fc receptor (FcRn),^26^ thereby accelerating catabolism of endogenous IgG. However, several observations from our study argue against this being the principal explanation for our findings. First, adenoviral antigen reactivity was enhanced in non-GBS individuals who received adenovectored vaccines (ChAdOx1 and Janssen) compared to those who received the mRNA-based Pfizer-BioNTech vaccine—none of whom had been exposed to IVIg—supporting the notion that the vector itself can elicit augmented anti-adenoviral responses. Second, among ChAdOx1 recipients, elevated antibody reactivity to a specific adenoviral antigen was present only in those who developed GBS, and not in those who did not, suggesting a targeted rather than polyclonal response pattern inconsistent with IVIg-related artefact. Third, direct comparison of adenoviral reactivity in samples taken in the presence or absence of prior IVIg administration revealed that differences in individual antigen signals were minor, not systematic, not restricted to a specific virus, and did not affect the adenoviral antigens most relevant to our main findings. Average signal intensity across the array was comparable between groups, and no significant differences were found for any of the key adenoviral targets. Collectively, these findings argue against IVIg being a major confounder in the observed adenoviral antibody responses associated with GBS.

Whilst we did not initially set out to look for an association in GBS overall, adenoviruses have previously been implicated in its pathogenesis. Terryberry *et al*.^27^ described adenoviral antibodies in 52% of patients with GBS, but this was not replicated by Jacobs *et al*.^28^ in a later study. However, a significant number of GBS cases was reported in temporal association with adenovirus vaccinations in the US military between 2011 and 2018.^29^ A UK-based prospective surveillance study^30^ identified 67 cases of GBS following ChAdOx1 vaccination and reported a higher-than-expected frequency of bilateral facial weakness with paraesthesias, along with more frequent sensory dysfunction compared to data from the International GBS Outcome Study (IGOS). In the same study, most cases occurred after the first vaccine dose, and more than one patient had identified antecedent infections. Similarly, and consistent with previous reports, all GBS-AV cases in our cohort followed the first dose of adenovectored vaccination. Only one individual in our cohort had a diarrhoeal illness 4 weeks prior to GBS onset, suggesting a potential alternative trigger. We did not observe an increased frequency of facial diplegia in the GBS-AV group compared to GBS-AC or GBS-NC. Given the high background seroprevalence of adenoviral antibodies, future studies may require quantitative or semi-quantitative assays able to differentiate antibodies to viral subunits to demonstrate significant differences.

How adenovirus might trigger GBS remains to be explained. Current thinking partitions GBS as a predominantly humoral disease, but antibodies to defined viral proteins have seldom been described. A recent study found autoreactive CD4+ and CD8+ memory T cells targeting P0, P2 or PMP22 in blood, CSF and nerve tissue of patients with GBS.^11^ That study included patients with GBS after SARS-CoV-2 infection. Very few had autoreactive T cells targeting myelin antigens, none of which cross-recognized SARS-CoV-2. Importantly, in the same study reactivity to cytomegalovirus (CMV) was explored because of previous described links. Some GBS patients with acute inflammatory demyelinating polyradiculoneuropathy (AIDP) had P0- and P2-specific T cell clones cross-reactive with CMV antigens, and vice versa some CMV-specific clones (from demyelinating GBS with previous CMV infection) cross-reacted with P0 and P2. This provided compelling evidence that preceding viral infection might induce T-cell-mediated peripheral nerve autoimmune demyelination. This was the nearest we got to a direct immunological link between GBS and a virus, but in that study, clones reactive to adenovirus were not sought. This should be a target of further research. Although humoral mechanisms remain possible, only 6.7% of GBS-AV patients had ganglioside antibodies—fewer than in GBS-AC and non-COVID-19-related GBS—suggesting that, if present, pathogenic antibodies in these cases may target other, as yet unidentified, antigens or clusters of antigens not routinely tested for. Similarly, 10% of GBS-AC patients had antibodies against nodal or paranodal proteins, and, despite otherwise meeting diagnostic criteria for GBS, these cases may in fact represent autoimmune nodopathies. In the co-cultures, we observed that sera from some GBS-AV patients reacted against myelin—a finding not seen in healthy or convalescent COVID-19 controls—raising the possibility that these antibodies may be pathogenically relevant. Although similar myelin reactivity was also detected in some GBS-NC patients, its presence in the GBS-AV group strengthens the case for humoral autoimmunity in vaccine-associated GBS.

The adenoviral array finding of elevated antibody reactivity to HAdV5-DNA-BP in post-vaccine GBS patients compared to uncomplicated ChAdOx1 vaccine recipients opens several avenues for further investigation. Both groups received the same adenovirus-vectored vaccine, yet only the former developed GBS and showed enhanced reactivity to this specific protein, suggesting its potential involvement in the pathogenesis of GBS. HAdV5-DNA-BP shows partial homology to the DNA binding protein of chimpanzee adenovirus Y25, used as the ChAdOx1 vaccine vector. A BLASTp alignment (query: YP_006272965.1; subject: UniProtKB P03265) revealed 54% identity and 68% similarity across the aligned regions, with only 5% gaps, indicating some conservation between the two proteins.^31^ No other targets with higher homology were included in the array. Despite this homology, the absence of high antibody reactivity in uncomplicated ChAdOx1 vaccine recipients indicates that the vaccine alone may not be sufficient to elicit humoral cross-reaction with HAdV5-DNA-BP, and that individual predisposition to autoimmunity as well as GBS-related mechanisms are involved. This is overall consistent with our finding of higher antibody reactivity against the S1 protein and adenoviruses in GBS-AC compared to uncomplicated controls, suggesting that such ‘enhanced’ responses may reflect underlying immunobiological dysfunctions in patients who develop GBS following COVID-19 infection or vaccination, absent in their uncomplicated post-infection or post-vaccination counterparts. Ultimately, the response to vaccination or infection may depend on the interaction between genetic, epigenetic and environmental factors. Finally, HAdV5-DNA-BP shows some degree of structural homology with several ubiquitously expressed intracellular proteins, including matrix proteins, zinc-finger and others. In GBS, these proteins may become (albeit non-pathogenically as intracellular) antigenic targets when nerves are damaged, inducing cross-reactivity with the structurally similar HAdV5-DNA-BP, not seen in individuals without GBS. These hypotheses warrant further investigation, and additional research is necessary to substantiate their validity.

### Limitations

This study has several limitations. It was facilitated by COVID-19 as a new viral infection, concerns about GBS as a result, and the rapid implementation of a novel vaccination strategy using adenoviral vectored and other vaccines with alternative delivery mechanisms. Such unique circumstances limit the opportunity to collect new samples or directly replicate the study. However, it provides evidence for an association between immunoreactivity against specific adenoviral antigens and GBS. Ascertainment bias and selection bias are major confounds. Several of the samples were likely sent for testing in view of an identified, close temporal correlation between COVID-19 and GBS onset and a heightened interest in this possible association. This may explain the significant difference between infection rate in our samples and background rate in England in the same period. As such, our cohort may not be representative of the whole GBS population at the time, and this precludes confident epidemiological inferences.

Collecting samples in the midst of the pandemic was difficult, and our groups were not originally intended for a prospectively designed study. The hypothesis emerged only later, following the occurrence of vaccination-induced GBS cases. There were several regrettable heterogeneities, including variations in sampling times, inconsistent access to clinical data, slightly unmatched cohorts, and the relatively small sample sizes. Small studies are prone to identify false positive results, but our results neatly fit the pre-defined hypothesis which emerged from GBS cases associated with adenoviral vectored COVID-19 vaccinations.^5^ Prospectively designed, fully powered, quantitative and mechanistic studies will be needed to carefully explore our assertion that adenoviruses may well be a common provoking infection for GBS.

## Conclusions

In conclusion, GBS following SARS-CoV-2 infection or vaccination does not have distinct clinical or serological features when compared to GBS overall, and each group is internally heterogenous. Despite a definite prodromal infection, the SARS-CoV-2 virus does not elicit an identifiable, homogenous humoral immune response against peripheral nerve antigens. Evidence of prior adenovirus infection can be found in patients with GBS regardless of previous COVID-19 infection or vaccination, with higher reactivity to human adenoviral proteins compared to uncomplicated vaccine controls. This suggests that adenoviruses may be causally associated with GBS and might account for some of the currently unexplained GBS cases. Post-vaccine GBS patients also have a lower incidence of ganglioside and paranodal antibodies, and a higher frequency of myelin-reactive antibodies, raising the possibility of a novel antigen association for this group. The observation that some patients with GBS have circulating antibodies capable of inducing complement-mediated demyelination *in vitro* also warrants further investigation. Nevertheless, this study finds no specific immunological signature underlying GBS following COVID-19 infection or vaccination but reveals adenoviruses as a potential contributor to GBS pathogenesis, paving the way for new avenues of research.

## Supplementary Material

awaf376_Supplementary_Data

## Data Availability

The data that support the findings of this study are available from the corresponding author, upon reasonable request.
