# Supplementary material for: Enhanced adenoviral reactivity in Guillain-Barré syndrome after SARS-CoV-2 infection and vaccination
Source: Brain. 2025 Oct 7;149(5):1718–31. doi: 10.1093/brain/awaf376 (PMC13140502; doi:10.1093/brain/awaf376)
Supplement: awaf376_Supplementary_Data [file awaf376_supplementary_data.pdf]

SUPPLEMENTARY APPENDIX

Contents **Page**

Supplementary Figures

Supplementary Figure 1: **Age and gender of patients and controls** **2**

Supplementary Figure 2: **Temporal intervals in GBS and controls** **3**

Supplementary Figure 3: **Clinical features of GBS patients** **4**

Supplementary Figure 4: **Frequency of SARS-CoV-2 infection in GBS and chronic neuropathy patients compared to national data in the early phase of the pandemic** **5**

Supplementary Figure 5: **Correlation between time from infection/vaccination to blood sample collection and ELISA IgG immunoreactivity** **6**

Supplementary Figure 6: **Immunoreactivity to gangliosides, nodal/paranodal antigens and myelinating co-cultures.** **7**

Supplementary Figure 7: **Electrochemiluminescent (ECL) cytokine profile analysis of GBS patients and controls.** **8**

Supplementary Figure 8: **Comparison of adenoviral reactivity in post-IVIg and non-IVIg serum samples.** **9**

Supplementary Tables

Supplementary Table 1: **Summary of included and excluded GBS patients** **10**

Supplementary Table 2: **Summary of adenoviral array comparisons** **13**

Supplementary Table 3: **Adenoviral antigens included on array** **18**

Supplementary Figure 1

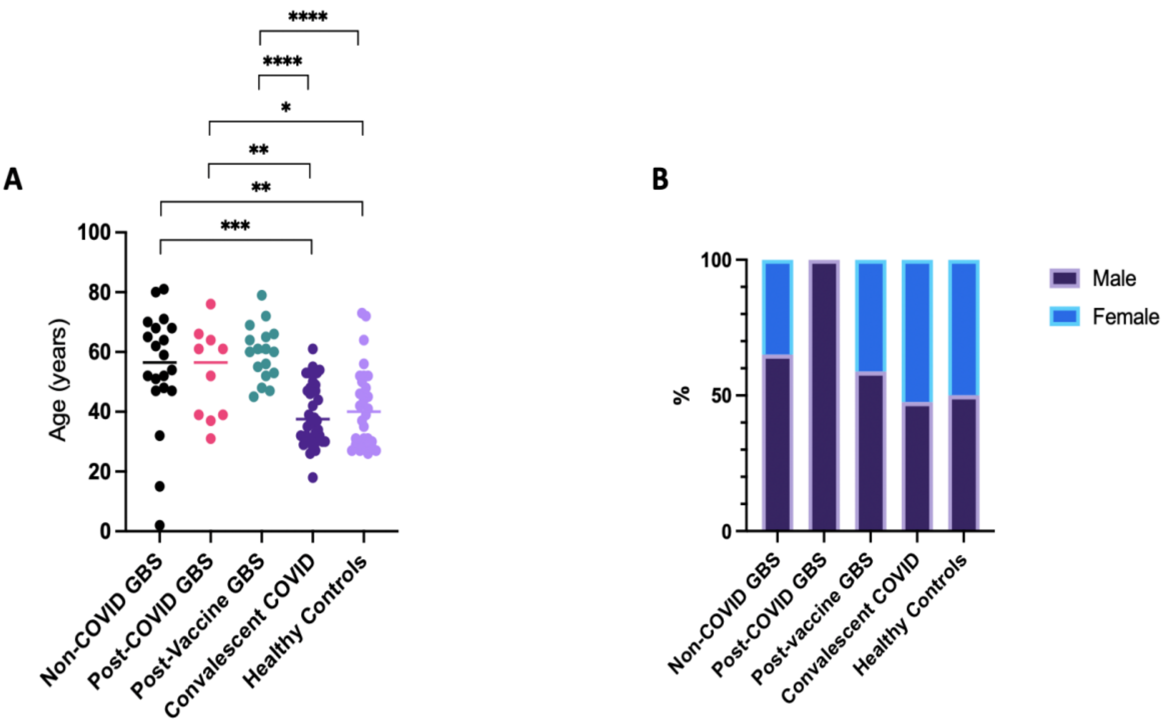

Supplementary Fig. 1: Age and gender of patients and controls. (A) Age and (B) gender of GBS patients without prior COVID infection or vaccination (Non-COVID GBS), after COVID infection (Post-COVID GBS), after COVID vaccination (Post-Vaccine GBS), convalescent controls, and healthy controls. * $P < 0.05$; ** $P < 0.01$; *** $P < 0.001$

Supplementary Figure 2

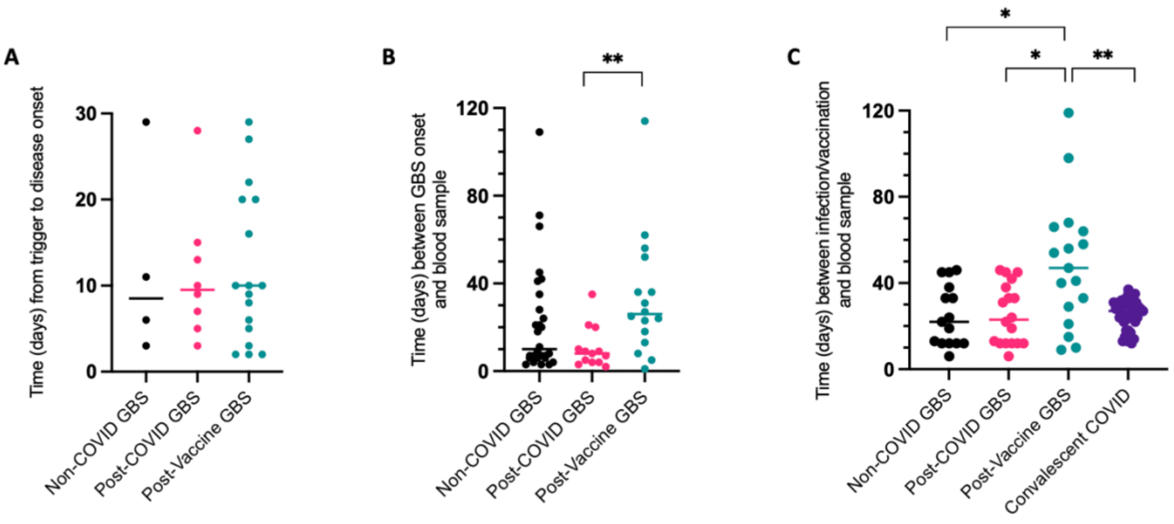

Supplementary Fig. 2: Temporal intervals in GBS and controls. (A) Time (days) between infectious trigger to disease onset; **(B)** days between GBS onset to blood sample collection; **(C)** days between infection or vaccination and blood sample collection. * *P < 0.05; ** P < 0.01; *** P < 0.001; **** P < 0.0001*

Supplementary Figure 3

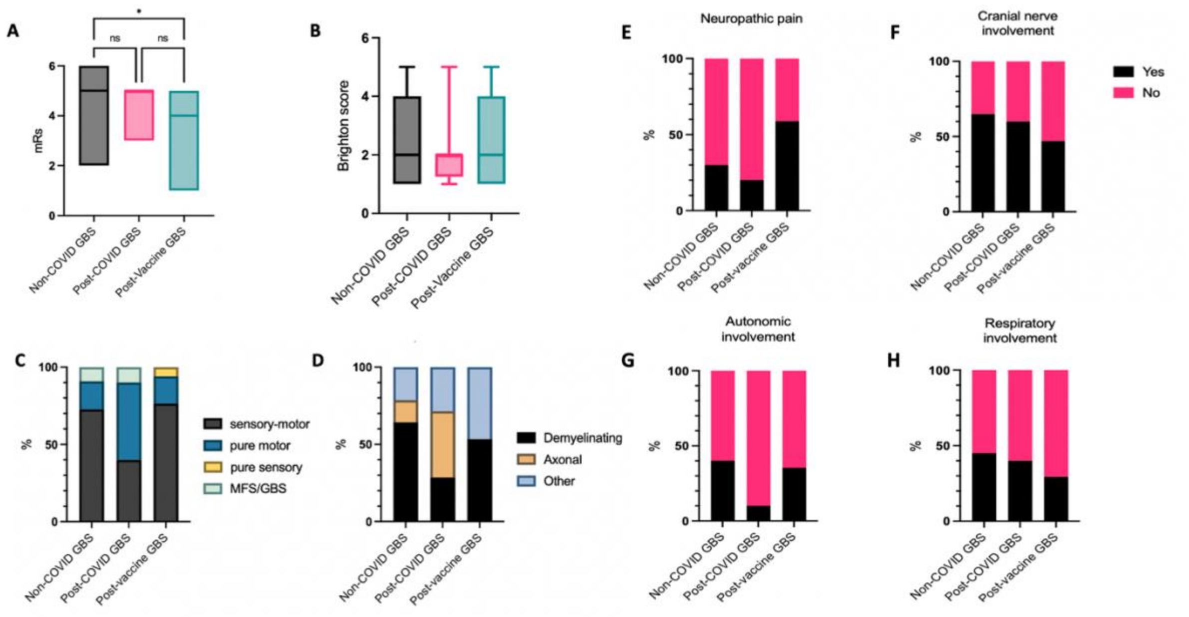

Supplementary Fig. 3: Clinical features of GBS patients. (A) Nadir disability of GBS patients. **(B)** Level of diagnostic certainty based on Brighton criteria with median and inter-quartile range indicated. Cases with a score of 5 had an alternative diagnosis and were excluded from further analysis. **(C)** Clinical subtype of GBS. **(D)** Electrophysiological classification of GBS. Other = equivocal / normal / inexcitable. **(E-F)** Fractions of GBS patients with neuropathic pain **(E)**, cranial neuropathies **(F)**, autonomic dysfunction **(G)**, respiratory involvement **(H)**. * *P = 0.04 by* Kruskal-Wallis/Dunn's, comparisons between GBS groups for all other parameters were non-significant.

Supplementary Figure 4

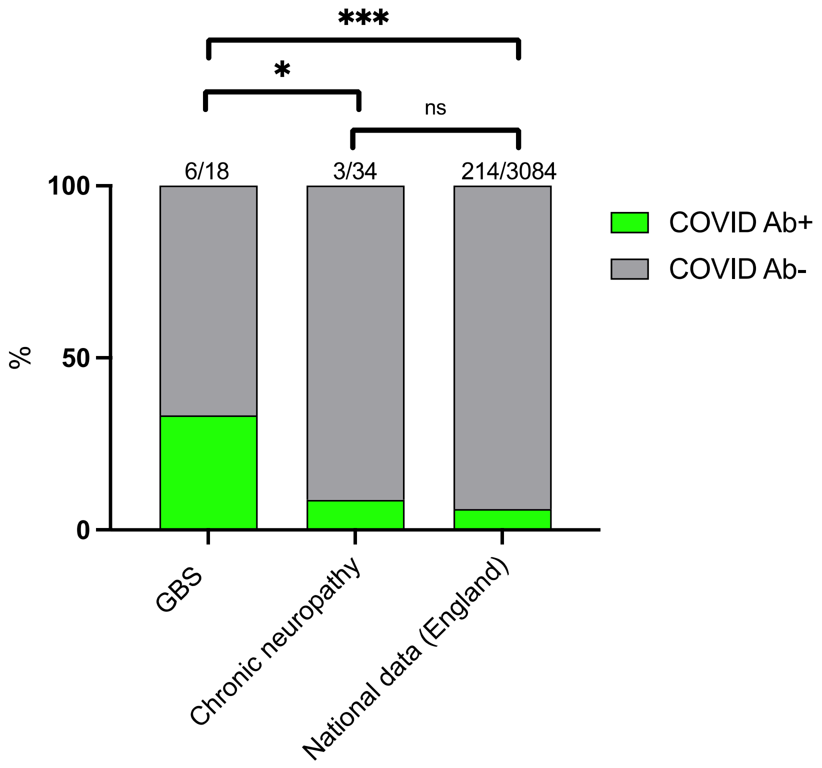

Supplementary Fig. 4: **Frequency of SARS-CoV-2 infection in GBS and chronic neuropathy patients compared to national data in the early phase of the pandemic.** GBS patients include only those meeting Brighton diagnostic criteria. * *P = 0.05* ***; *P < 0.001* (Fisher's exact)

Supplementary Figure 5

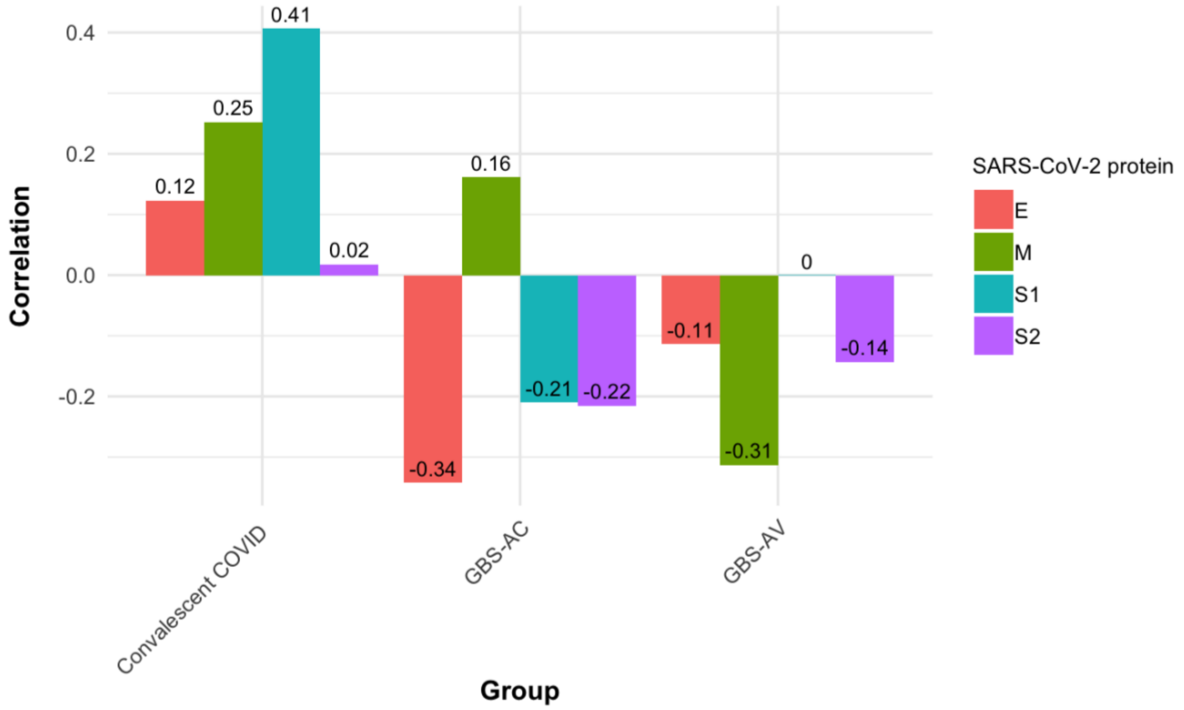

Supplementary Fig. 5: **Correlation between time from infection/vaccination to blood sample collection and ELISA IgG immunoreactivity.** The latter was assessed against SARS-CoV-2 envelope (E), membrane (M) and spike (S1, S2) proteins in sera from GBS patients with previous COVID (GBS-AC), vaccination (GBS-AV) and convalescent COVID controls (without GBS).

Supplementary Figure 6

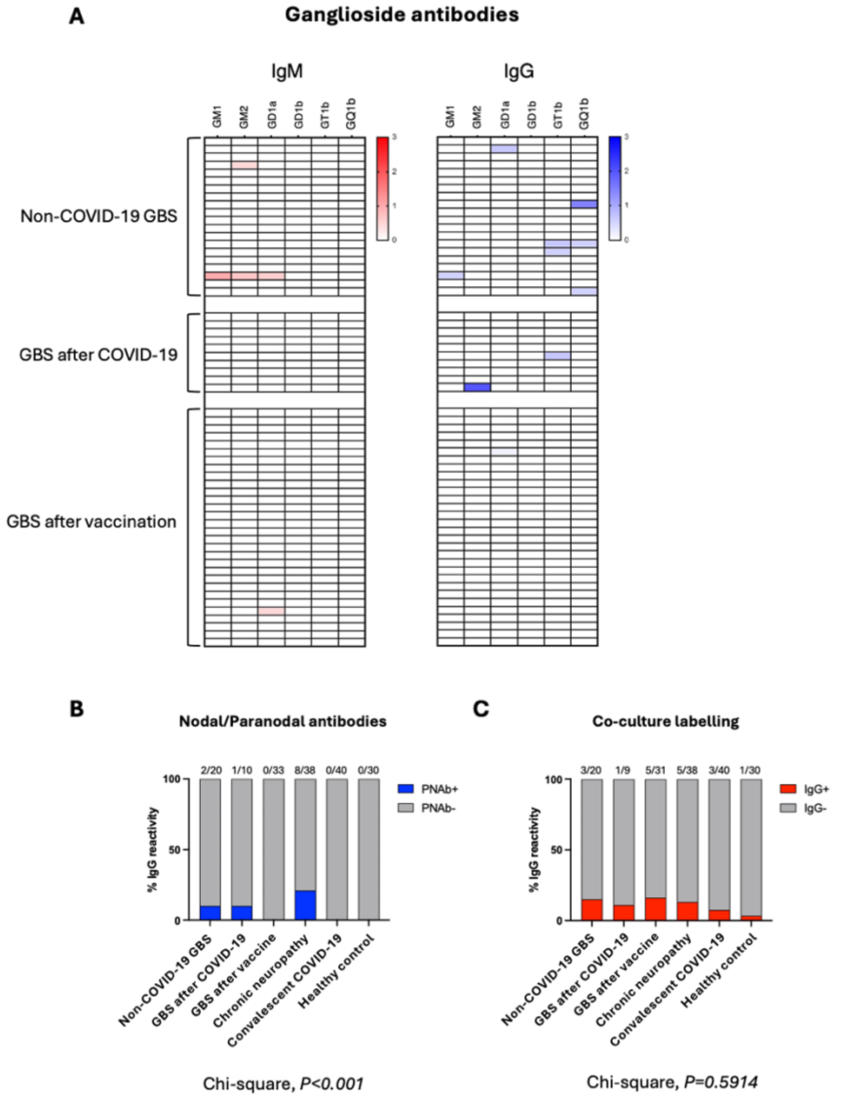

Supplementary Fig. 6: Immunoreactivity to gangliosides, nodal/paranodal antigens and myelinating co-cultures. (A) Ganglioside antibodies were more frequent in non-COVID-19 GBS (7/20, 35%) compared to GBS after COVID-19 (2/10, 20%) and GBS after vaccination (2/30, 6.7%), but these differences were not statistically significant. IgG anti-ganglioside antibodies were observed in fewer GBS-AV patients compared to GBS-NC [*P = 0.01*, OR 0.08 (95% CI 0.01 to 0.63)]. **(B)** PNAbs frequency varies between groups (Chi-square, *P < 0.001*). **(C)** Myelinating co-culture labelling revealed no significant differences between groups (Chi-square, *P = 0.5914*).

Supplementary Figure 7

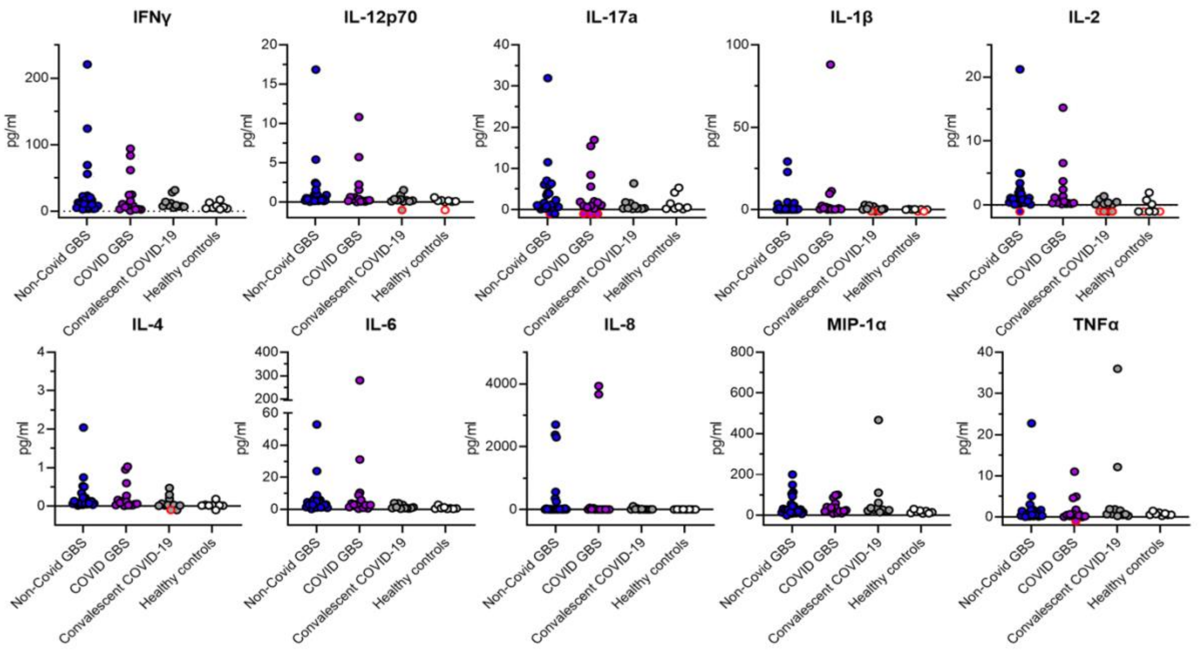

Supplementary Fig. 7: Electrochemiluminescent (ECL) cytokine profile analysis of GBS patients and controls. No differences were found between GBS groups, convalescent COVID and healthy controls. Red circles indicate samples with levels below assay detection limit.

Supplementary Figure 8

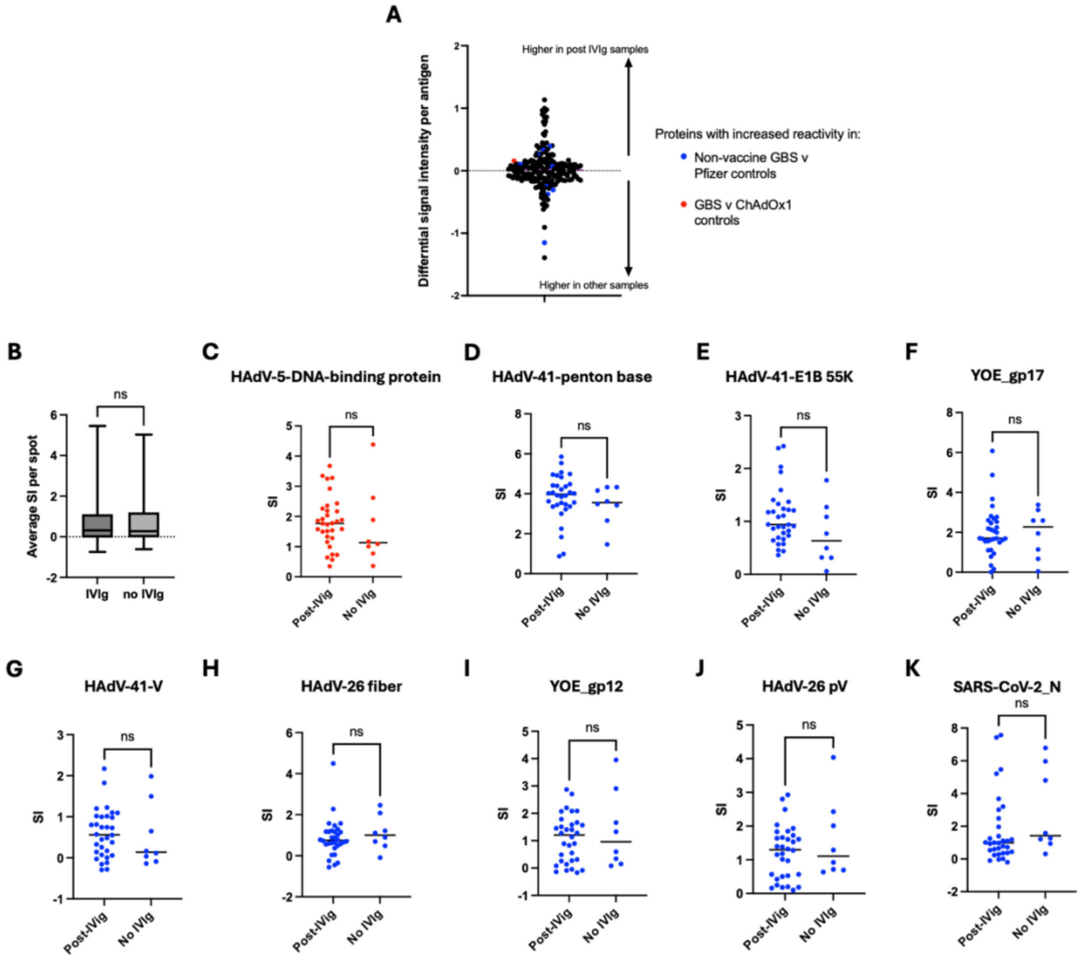

Supplementary Fig. 8: Comparison of adenoviral reactivity in post-IVIg and non-IVIg serum samples. (A) Difference in average signal intensity (SI) between post-IVIg and non-IVIg serum samples from patients with GBS. The data point in red indicates the protein (HAdV-5 DNA binding protein) which produced significantly increased reactivity with GBS v uncomplicated ChAdOX1 sera. The data points in blue reflect proteins producing significant enhanced reactivity in non-vaccine GBS compared to uncomplicated Pfizer-BioNTech sera. **(B)** There was no significant difference in average SI per spot between the post-IVIg and non-IVIg groups. **(C-K)** Difference in average SI between post-IVIg and non-IVIg was also evaluated for other proteins which produced increased reactivity in GBS patients v uncomplicated vaccine recipients.

Supplementary Table 1: Summary of included and excluded GBS patients

Patient	GBS group	Brighton Score	Included / excluded	Reason for exclusion / score >1	Evidence for recent COVID infection	
					PCR	Serology
1	NC-GBS	1	Included			
2	NC-GBS	4	Included	Subacute onset		
3	NC-GBS	1	Included			
4	NC-GBS	1	Included			
5	NC-GBS	1	Included			
6	NC-GBS	1	Included			
7	NC-GBS	2	Included	No NCS		
8	NC-GBS	2	Included	No CSF		
9	NC-GBS	2	Included	No NCS		
10	NC-GBS	Not met	Excluded	CIDP		
11	NC-GBS	4	Included	Subacute onset		
12	NC-GBS	1	Included			
13	NC-GBS	3	Included	No NCS, normal CSF protein		
14	NC-GBS	3	Included	No NCS/CSF		
15	NC-GBS	4	Included	Subacute onset, normal NCS, no CSF		
16	NC-GBS	1	Included			
17	NC-GBS	3	Included	CSF normal, NCS equivocal		
18	NC-GBS	2	Included	CSF normal		
19	NC-GBS	1	Included			
20	NC-GBS	1	Included			
21	NC-GBS	Not met	Excluded	CIDP		
22	NC-GBS	Not met	Excluded	CIDP		

23	NC-GBS	1	Included			
24	GBS-AC	2	Included	No NCS	NEG	POS
25	GBS-AC	1	Included		NOT DONE	POS
26	GBS-AC	2	Included		NEG	POS
27	GBS-AC	Not met	Excluded	BC not met; transverse myelitis	NEG	POS
28	GBS-AC	2	Included	No NCS	NEG	POS
29	GBS-AC	1	Included		POS	NEG
30	GBS-AC	2	Included	Equivocal NCS	POS	POS
31	GBS-AC	Not met	Excluded	BC not met; thiamine deficiency	POS	POS
32	GBS-AC	1	Included		POS	POS
33	GBS-AC	2	Included	No CSF	POS	POS
34	GBS-AC	2	Included	CSF normal	POS	NEG
35	GBS-AC	2	Included	No NCS	NEG	POS
36	GBS-AV	2	Included	No CSF		
37	GBS-AV	1	Included			
38	GBS-AV	2	Included	No CSF		
39	GBS-AV	1	Excluded	<0 days		
40	GBS-AV	1	Included			
41	GBS-AV	4	Included	Subacute onset		
42	GBS-AV	2	Included	No NCS		
43	GBS-AV	1	Included			
44	GBS-AV	1	Excluded	>42 days		
45	GBS-AV	1	Excluded	>42 days		
46	GBS-AV	1	Included			
47	GBS-AV	1	Included			
48	GBS-AV	1	Included			
49	GBS-AV	4	Included	Brisk reflexes, normal NCS		
50	GBS-AV	4	Included	Subacute onset		

51	GBS-AV	4	Included	Initially brisk reflexes		
52	GBS-AV	4	Included	Preserved reflexes		
53	GBS-AV	1	Included			
54	GBS-AV	Not met	Excluded	CIDP		
55	GBS-AV	4	Included	Subacute progression, normal CSF protein		
56	GBS-AV	4	Included	Pure sensory, normal CSF protein, equivocal NCS		
57	GBS-AV	Not met	Excluded	CIDP		
58	GBS-AV	Not met	Excluded	CIDP		
59	GBS-AV	3	Included	Normal CSF protein, equivocal NCS		

NC-GBS: non-COVID GBS; GBS-AC: GBS after COVID infection; GBS-AV: GBS after vaccination; NCS, nerve conduction studies; CSF, cerebrospinal fluid; CIDP, chronic inflammatory demyelinating polyradiculoneuropathy; BC, Brighton Criteria.

Supplementary Table 2: Summary of adenoviral array comparisons

GBS group	Uncomplicated vaccine group	Significance	Significant antibody (P value)
GBS-AC	ChAdOx1		
GBS-AC	Janssen	Significant	HAdV-41-E2A DBP (0.03518765)<br>YOE_gp17 (0.03683076)<br>HAdV-41-E1A (0.04632693)
GBS-AC	Pfizer-BioNTech	Significant	HAdV-41-penton base (0.03452916)<br>HAdV-40-L2 penton base (0.03737796)<br>HAdV-5-protein VI precursor (0.04853187)<br>YOE_gp17 (0.04853187)<br>HAdV-26 100K shutoff protein (0.04853187)<br>SARS-CoV-2_USA-WA1/2020-N (0.04853187)<br>HAdV-4-L3 protein VI (0.04853187)<br>HAdV-41-E1A (0.04853187)
GBS-AC	ChAdOx1 + Janssen		
GBS-AC	Janssen + Pfizer-BioNTech	Significant	HAdV-41-E2A DBP (0.01884876)<br>YOE_gp17 (0.01884876)<br>HAdV-4-early E2A DNA-binding protein (0.04989042)<br>HAdV-41-E1A (0.04672054)
GBS-AC	ChAdOx1 + Pfizer-BioNTech		
GBS-AC	ChAdOx1 + Janssen + Pfizer-BioNTech		
GBS-AV	ChAdOx1	Significant	HAdV-5-DNA-binding protein (0.03508039)
GBS-AV	Janssen		

GBS-AV	Pfizer-BioNTech		
GBS-AV	ChAdOx1 + Janssen		
GBS-AV	Janssen + Pfizer-BioNTech		
GBS-AV	ChAdOx1 + Pfizer-BioNTech		
GBS-AV	ChAdOx1 + Janssen + Pfizer-BioNTech		
GBS-NC	ChAdOx1		
GBS-NC	Janssen		
GBS-NC	Pfizer-BioNTech		
GBS-NC	ChAdOx1 + Janssen		
GBS-NC	Janssen + Pfizer-BioNTech		
GBS-NC	ChAdOx1 + Pfizer-BioNTech		
GBS-NC	ChAdOx1 + Janssen + Pfizer-BioNTech		
GBS-AC + GBS-AV	ChAdOx1	Significant	HAdV-5-DNA-binding protein (0.002034706)
GBS-AC + GBS-AV	Janssen	Significant	HAdV-41-E2A DBP (0.00055463)<br>YOE_gp17 (0.00036784)<br>HAdV-4-early E2A DNA-binding protein (0.00029750)<br>HAdV-41-E1A (0.00044979)
GBS-AC + GBS-AV	Pfizer-BioNTech	Significant	HAdV-4-L3 protein VI (0.005870831)<br>HAdV-4-E4 13.7 kDa protein (0.028269730)
GBS-AC + GBS-AV	ChAdOx1 + Janssen		
GBS-AC + GBS-AV	Janssen + Pfizer-BioNTech	Significant	HAdV-41-E2A DBP (0.01366655)<br>YOE_gp17 (0.01018427)<br>HAdV-4-early E2A DNA-binding protein (0.01018427)<br>HAdV-41-E1A (0.01018427)
GBS-AC + GBS-AV	ChAdOx1 + Pfizer-BioNTech		
GBS-AC + GBS-AV	ChAdOx1 + Janssen + Pfizer-BioNTech		

GBS-AV + GBS-NC	ChAdOx1	Significant	HAdV-5-DNA-binding protein (0.002012703)
GBS-AV + GBS-NC	Janssen	Significant	HAdV-41-E2A DBP (0.0456087)<br>YOE_gp17 (0.0456087)<br>HAdV-4-early E2A DNA-binding protein (0.0456087)
GBS-AV + GBS-NC	Pfizer-BioNTech	Significant	HAdV-4-L3 protein VI (0.03491937)
GBS-AV + GBS-NC	ChAdOx1 + Janssen		
GBS-AV + GBS-NC	Janssen + Pfizer-BioNTech	Significant	HAdV-41-E2A DBP (0.03593143)<br>YOE_gp17 (0.03593143)<br>HAdV-4-early E2A DNA-binding protein (0.03593143)<br>HAdV-41-E1A (0.04693881)
GBS-AV + GBS-NC	ChAdOx1 + Pfizer-BioNTech		
GBS-AV + GBS-NC	ChAdOx1 + Janssen + Pfizer-BioNTech		
GBS-AC + GBS-NC	ChAdOx1	Significant	HAdV-5-DNA-binding protein (0.008618702)
GBS-AC + GBS-NC	Janssen	Significant	HAdV-41-E2A DBP (0.03334759)<br>HAdV-40-E2A-L single-stranded DNA-binding protein (0.03533542)<br>YOE_gp17 (0.03334759)<br>HAdV-4-early E2A DNA-binding protein (0.03334759)<br>HAdV-41-E1A (0.03334759)

GBS-AC + GBS-NC	Pfizer-BioNTech	Significant	HAdV-41-penton base (0.03933281)<br>YOE_gp17 (0.04701653)<br>SARS-CoV-2_USA-WA1/2020-N (0.03933281)<br>HAdV-4-L3 protein VI (0.01690616)<br>HAdV-26 pV (0.01954619)<br>YOE_gp12 (0.01954619)<br>HAdV-41-E1B 55K (0.03582921)<br>HAdV-26 fiber (0.03933281)<br>HAdV-41-V (0.03933281)
GBS-AC + GBS-NC	ChAdOx1 + Janssen		
GBS-AC + GBS-NC	Janssen + Pfizer-BioNTech	Significant	HAdV-41-E2A DBP (0.02430272)<br>HAdV-40-E2A-L single-stranded DNA-binding protein (0.02430272)<br>YOE_gp17 (0.02430272)<br>HAdV-4-early E2A DNA-binding protein (0.02433199)<br>HAdV-41-E1A (0.02430272)
GBS-AC + GBS-NC	ChAdOx1 + Pfizer-BioNTech		
GBS-AC + GBS-NC	ChAdOx1 + Janssen + Pfizer-BioNTech		
All GBS	ChAdOx1	Significant	HAdV-5-DNA-binding protein (0.0001216797)
All GBS	Janssen	Significant	HAdV-41-E2A DBP (0.005536673)<br>HAdV-40-E2A-L single-stranded DNA-binding protein (0.047776892)<br>YOE_gp17 (0.005536673)<br>HAdV-4-early E2A DNA-binding protein (0.005536673)<br>HAdV-41-E1A (0.010901705)

All GBS	Pfizer-BioNTech	Significant	HAdV-41-penton base (0.0408883950)<br>HAdV-40-L2 penton base (0.0408883950)<br>YOE_gp17 (0.0438935702)<br>SARS-CoV-2_USA-WA1/2020-N (0.0408883950)<br>HAdV-4-L3 protein VI (0.0008769344)<br>HAdV-26 pV (0.0438935702)<br>YOE_gp12 (0.0408883950)<br>HAdV-41-E1B 55K (0.0408883950)<br>HAdV-26 fiber (0.0408883950)<br>HAdV-4-E4 13.7 kDa protein (0.0374388616
All GBS	ChAdOx1 + Janssen		
All GBS	Janssen + Pfizer-BioNTech	Significant	HAdV-41-E2A DBP (0.004016131)<br>HAdV-40-E2A-L single-stranded DNA-binding protein (0.022911602)<br>YOE_gp17 (0.004016131)<br>HAdV-4-early E2A DNA-binding protein (0.004016131)<br>HAdV-41-E1A (0.004016131)
All GBS	ChAdOx1 + Pfizer-BioNTech		
All GBS	ChAdOx1 + Janssen + Pfizer-BioNTech		
GBS-AV	NC-GBS		
GBS-AC	NC-GBS		
GBS-AV	GBS-AC		
GBS-AC + GBS-AV	NC-GBS		
GBS-AC + GBS-NC	GBS-AV		
GBS-AV + GBS-NC	GBS-AC		

Supplementary table 3: Adenoviral antigens included on array

Virus	Antigen	Included in final analysis
**HAdV26**	HAdV-26 100K shutoff protein	Y
**HAdV26**	HAdV-26 52/55K packaging protein 3	Y
**HAdV26**	HAdV-26 DNA binding protein	Y
**HAdV26**	HAdV-26 DNA polymerase	Y
**HAdV26**	HAdV-26 fiber	Y
**HAdV26**	HAdV-26 hexon	Y
**HAdV26**	HAdV-26 penton	Y
**HAdV26**	HAdV-26 pIIIa	Y
**HAdV26**	HAdV-26 pIIIa hexon-linking protein IIIa	Y
**HAdV26**	HAdV-26 pIX hexon-interlacing protein	N
**HAdV26**	HAdV-26 protease	N
**HAdV26**	HAdV-26 pTP intermediate terminal protein	N
**HAdV26**	HAdV-26 pTP terminal protein	N
**HAdV26**	HAdV-26 pTP_preterminal protein	N
**HAdV26**	HAdV-26 pV	Y
**HAdV26**	HAdV-26 pVI pre-protein VI	Y
**HAdV26**	HAdV-26 pVI protease cofactor	N
**HAdV26**	HAdV-26 pVII histone-like nucleoprotein	Y
**HAdV26**	HAdV-26 pVII pre-histone-like nucleoprotein	Y
**HAdV26**	HAdV-26 pVIII hexon-linking protein-C	N
**HAdV26**	HAdV-26 pVIII hexon-linking protein-N	N
**HAdV26**	HAdV-26 pVIII pre-hexon-linking protein VIII	Y
**HAdV4**	HAdV-4-11.5 kDa early protein	N
**HAdV4**	HAdV-4-14.1 kDa early protein	N
**HAdV4**	HAdV-4-19 kDa small T antigen	N
**HAdV4**	HAdV-4-19.4 kDa early protein	N
**HAdV4**	HAdV-4-52 kDa L1 protein	N
**HAdV4**	HAdV-4-DNA polymerase_s1_US	N
**HAdV4**	HAdV-4-DNA polymerase_s2	N
**HAdV4**	HAdV-4-DNA-binding protein	Y
**HAdV4**	HAdV-4-E3 10.4 kDa protein	N
**HAdV4**	HAdV-4-E3 12.1 kDa protein	N
**HAdV4**	HAdV-4-E3 14.5 kDa protein	N
**HAdV4**	HAdV-4-E3 14.7 kDa protein	N
**HAdV4**	HAdV-4-E3 19 kDa protein	N
**HAdV4**	HAdV-4-E3 23.3 kDa protein	Y
**HAdV4**	HAdV-4-E3 24.8 kDa protein	N
**HAdV4**	HAdV-4-E3 29.7 kDa protein	N
**HAdV4**	HAdV-4-E3 6.3 kDa protein	N
**HAdV4**	HAdV-4-E4 13.5 kDa protein	N
**HAdV4**	HAdV-4-E4 13.7 kDa protein	Y
**HAdV4**	HAdV-4-E4 14.1 kDa protein	N

**HAdV4**	HAdV-4-E4 14.6 kDa protein	N
**HAdV4**	HAdV-4-E4 15.9 kDa protein	N
**HAdV4**	HAdV-4-E4 34.6 kDa protein	N
**HAdV4**	HAdV-4-E4 7.4 kDa protein	N
**HAdV4**	HAdV-4-early E1A 24.6 kDa protein	N
**HAdV4**	HAdV-4-early E1A 28 kDa protein	Y
**HAdV4**	HAdV-4-early E1A 6.8 kDa protein	N
**HAdV4**	HAdV-4-early E1B 16.8 kDa protein	N
**HAdV4**	HAdV-4-early E1B 20 kDa protein	N
**HAdV4**	HAdV-4-early E1B 8.2 kDa protein	N
**HAdV4**	HAdV-4-early E2A DNA-binding protein	Y
**HAdV4**	HAdV-4-L1 protein IIIa	Y
**HAdV4**	HAdV-4-L2 penton protein	Y
**HAdV4**	HAdV-4-L2 protein X	Y
**HAdV4**	HAdV-4-L3 hexon protein_US	N
**HAdV4**	HAdV-4-L3 protein VI	Y
**HAdV4**	HAdV-4-L4 100 kDa protein_US	Y
**HAdV4**	HAdV-4-L4 33 kDa protein	Y
**HAdV4**	HAdV-4-L4 protein VIII	N
**HAdV4**	HAdV-4-large T antigen	N
**HAdV4**	HAdV-4-late L3 23 kDa proteinase	N
**HAdV4**	HAdV-4-ORF1 protein	N
**HAdV4**	HAdV-4-protein IX	Y
**HAdV40**	HAdV-40-E1A control protein E1A	Y
**HAdV40**	HAdV-40-E1B control protein E1B 19K	N
**HAdV40**	HAdV-40-E1B control protein E1B 55K	N
**HAdV40**	HAdV-40-E2A-L single-stranded DNA-binding protein	Y
**HAdV40**	HAdV-40-E2B DNA polymerase_s1-1	N
**HAdV40**	HAdV-40-E2B DNA polymerase_s2	N
**HAdV40**	HAdV-40-E2B DNA polymerase-10	N
**HAdV40**	HAdV-40-E2B terminal protein precursor pTP	Y
**HAdV40**	HAdV-40-E3A membrane glycoprotein E3 CR1-alpha	N
**HAdV40**	HAdV-40-E3A membrane glycoprotein E3 CR1-beta	Y
**HAdV40**	HAdV-40-E3B control protein E3 14.7K	N
**HAdV40**	HAdV-40-E3B membrane protein E3 RID-alpha	N
**HAdV40**	HAdV-40-E3B membrane protein E3 RID-beta	N
**HAdV40**	HAdV-40-E4 control protein E4 34K	N
**HAdV40**	HAdV-40-E4 control protein E4orf2	N
**HAdV40**	HAdV-40-E4 control protein E4orf3	N
**HAdV40**	HAdV-40-E4 control protein E4orf4	N
**HAdV40**	HAdV-40-E4 control protein E4orf6/7	N
**HAdV40**	HAdV-40-IVa2 encapsidation protein IVa2	Y
**HAdV40**	HAdV-40-IX capsid protein IX	N
**HAdV40**	HAdV-40-L1 capsid protein precursor pIIIa	Y
**HAdV40**	HAdV-40-L1 encapsidation protein 52K	Y
**HAdV40**	HAdV-40-L2 core protein precursor pVII	Y
**HAdV40**	HAdV-40-L2 core protein precursor pX	N

**HAdV40**	HAdV-40-L2 core protein V	Y
**HAdV40**	HAdV-40-L2 penton base	Y
**HAdV40**	HAdV-40-L3 capsid protein precursor pVI	Y
**HAdV40**	HAdV-40-L3 hexon	N
**HAdV40**	HAdV-40-L3 protease	N
**HAdV40**	HAdV-40-L4A capsid protein precursor pVIII	Y
**HAdV40**	HAdV-40-L4A encapsidation protein 22K	Y
**HAdV40**	HAdV-40-L4A hexon assembly protein 100K	Y
**HAdV40**	HAdV-40-L4A protein 33K	Y
**HAdV40**	HAdV-40-L5 fiber	Y
**HAdV40**	HAdV-40-L5A fiber-2	Y
**HAdV40**	HAdV-40-U protein U	Y
**HAdV41**	HAdV-41-100K	Y
**HAdV41**	HAdV-41-52-55K	Y
**HAdV41**	HAdV-41-E1A	Y
**HAdV41**	HAdV-41-E1B 19K	N
**HAdV41**	HAdV-41-E1B 55K	Y
**HAdV41**	HAdV-41-E2A DBP	Y
**HAdV41**	HAdV-41-E2B DNA pol_s1	N
**HAdV41**	HAdV-41-E2B DNA pol_s2	N
**HAdV41**	HAdV-41-E2B DNA pol-4	N
**HAdV41**	HAdV-41-E2B preterminal protein	Y
**HAdV41**	HAdV-41-E3 10.1K	Y
**HAdV41**	HAdV-41-E3 14.5K	N
**HAdV41**	HAdV-41-E3 14.7K	N
**HAdV41**	HAdV-41-E3 19.4K	N
**HAdV41**	HAdV-41-E3 31.6K	Y
**HAdV41**	HAdV-41-E4 hypothetical protein 2	N
**HAdV41**	HAdV-41-E4 hypothetical protein 3	N
**HAdV41**	HAdV-41-E4 hypothetical protein 4	N
**HAdV41**	HAdV-41-E4 hypothetical protein 6	N
**HAdV41**	HAdV-41-hexon	N
**HAdV41**	HAdV-41-i-leader protein	Y
**HAdV41**	HAdV-41-IIIa	Y
**HAdV41**	HAdV-41-IVa2	N
**HAdV41**	HAdV-41-IX	N
**HAdV41**	HAdV-41-L2 pMu	N
**HAdV41**	HAdV-41-long fiber protein	Y
**HAdV41**	HAdV-41-penton base	Y
**HAdV41**	HAdV-41-protease	N
**HAdV41**	HAdV-41-pVII	Y
**HAdV41**	HAdV-41-pVIII	Y
**HAdV41**	HAdV-41-short fiber protein	Y
**HAdV41**	HAdV-41-truncated U exon protein	N
**HAdV41**	HAdV-41-V	Y
**HAdV41**	HAdV-41-VI	Y
**HAdV5**	HAdV-5-10.4 kDa protein	N

**HAdV5**	HAdV-5-10.5 kDa protein	Y
**HAdV5**	HAdV-5-12.5 kDa protein	Y
**HAdV5**	HAdV-5-13.3 kDa protein	N
**HAdV5**	HAdV-5-13.4 kDa protein	Y
**HAdV5**	HAdV-5-14.3 kDa protein	Y
**HAdV5**	HAdV-5-14.6 kDa protein	Y
**HAdV5**	HAdV-5-17 kDa protein	N
**HAdV5**	HAdV-5-18.5 kDa protein	N
**HAdV5**	HAdV-5-23 kDa proteinase	N
**HAdV5**	HAdV-5-26 kDa protein	Y
**HAdV5**	HAdV-5-32 kDa protein	Y
**HAdV5**	HAdV-5-33 kDa protein	Y
**HAdV5**	HAdV-5-34 kDa protein	N
**HAdV5**	HAdV-5-6 kDa protein	Y
**HAdV5**	HAdV-5-7 kDa protein	N
**HAdV5**	HAdV-5-7.2 kDa protein	N
**HAdV5**	HAdV-5-DNA polymerase_s2	N
**HAdV5**	HAdV-5-DNA-binding protein	Y
**HAdV5**	HAdV-5-E2A DNA-binding protein	N
**HAdV5**	HAdV-5-E3-14.7 kDa protein	N
**HAdV5**	HAdV-5-E4 11 kDa protein	N
**HAdV5**	HAdV-5-E4-14.7 kDa protein	N
**HAdV5**	HAdV-5-fiber protein	Y
**HAdV5**	HAdV-5-hexon protein	Y
**HAdV5**	HAdV-5-hexon-associated protein IX	N
**HAdV5**	HAdV-5-hypothetical 11.7 kDa early protein	N
**HAdV5**	HAdV-5-hypothetical 12 kDa early protein	N
**HAdV5**	HAdV-5-hypothetical 12.7 kDa protein	N
**HAdV5**	HAdV-5-hypothetical 12.8 early protein	N
**HAdV5**	HAdV-5-hypothetical 14.4 kDa protein	N
**HAdV5**	HAdV-5-penton protein_US	Y
**HAdV5**	HAdV-5-protein IIIa precursor	Y
**HAdV5**	HAdV-5-protein VI precursor	Y
**HAdV5**	HAdV-5-protein VIII	Y
**HAdV5**	HAdV-5-small T antigen	Y
**HAdV5**	HAdV-5-terminal protein precursor_US	Y
**SARS-CoV-2**	SARS-CoV-2_USA-WA1/2020-N	Y
**SARS-CoV-2**	SARS-CoV-2_USA-WA1/2020-S2	Y
**ChAdV Y25**	YOE_gp01-100kDa	Y
**ChAdV Y25**	YOE_gp02-22K	Y
**ChAdV Y25**	YOE_gp03-33K	N
**ChAdV Y25**	YOE_gp04-52/55kDa	N
**ChAdV Y25**	YOE_gp05-DNA binding protein	Y
**ChAdV Y25**	YOE_gp06-E1A	Y
**ChAdV Y25**	YOE_gp07-E1B 19K	Y
**ChAdV Y25**	YOE_gp08-E1B 55K	Y
**ChAdV Y25**	YOE_gp09-E3 12.5K	Y

**ChAdV Y25**	YOE_gp10-E3 14.7kDa	Y
**ChAdV Y25**	YOE_gp11-E3 gp19K	Y
**ChAdV Y25**	YOE_gp12-E4Orf1	Y
**ChAdV Y25**	YOE_gp13-E4Orf2	N
**ChAdV Y25**	YOE_gp14-E4Orf3	Y
**ChAdV Y25**	YOE_gp15-E4Orf4	N
**ChAdV Y25**	YOE_gp16-E4Orf6	N
**ChAdV Y25**	YOE_gp17-E4Orf6/7	Y
**ChAdV Y25**	YOE_gp18-endoprotease	Y
**ChAdV Y25**	YOE_gp19-fiber	Y
**ChAdV Y25**	YOE_gp20-hexon	Y
**ChAdV Y25**	YOE_gp21-IVa2	Y
**ChAdV Y25**	YOE_gp22-Mu	Y
**ChAdV Y25**	YOE_gp23-penton	Y
**ChAdV Y25**	YOE_gp24-pIIIa	N
**ChAdV Y25**	YOE_gp25-pIX	Y
**ChAdV Y25**	YOE_gp26-polymerase	N
**ChAdV Y25**	YOE_gp27-pTP	N
**ChAdV Y25**	YOE_gp28-pV	N
**ChAdV Y25**	YOE_gp29-pVI	N
**ChAdV Y25**	YOE_gp30-pVII	N
**ChAdV Y25**	YOE_gp31-pVIII	N
